# Dendritic cell circadian clocks shape memory CD8^+^ T cell differentiation

**DOI:** 10.1126/sciadv.aeh3719

**Published:** 2026-07-31

**Authors:** Ward Vleeshouwers, Suzanne van Duikeren, J. Fréderique de Graaf, Dominique M. B. Veerkamp, Marissa E. Linger, Sytze H. T. Jorritsma, Sebenzile K. Myeni, Laura Kervezee, Ramon Arens

**Affiliations:** ^1^ Department of Immunology, Leiden University Medical Center, Albinusdreef 2, 2333 ZA Leiden, The Netherlands.; ^2^ Leiden University Center for Infectious Diseases, Leiden University Medical Center, Albinusdreef 2, 2333 ZA Leiden, The Netherlands.; ^3^ Department of Cell and Chemical Biology, Leiden University Medical Center, Einthovenweg 20, 2333 ZC Leiden, The Netherlands.

## Abstract

Circadian rhythms regulate diverse immune processes, yet how they influence memory CD8^+^ T cell differentiation remains unclear. Here, we show that the time of day of antigen encounter shapes CD8^+^ T cell fate and antiviral immunity. Immunization during the active phase promotes the generation of progenitor-like memory CD8^+^ T cells and enhances T cell-mediated protection upon viral challenge. Mechanistically, dendritic cell-intrinsic circadian clocks regulate expression of the costimulatory ligand CD70, thereby directing T cell differentiation. These findings uncover a dendritic cell-mediated circadian mechanism that governs memory T cell fate decisions and suggest that aligning immune priming with circadian time may be leveraged to optimize T cell immunity.

## Teaser

Circadian timing of antigen exposure programs CD8^+^ T cell memory and tunes antiviral immunity.

## INTRODUCTION

The generation of durable memory CD8^+^ T cell responses is a central goal of vaccination and immunotherapy. Following antigen encounter, naïve CD8^+^ T cells undergo robust expansion and differentiation, giving rise to heterogeneous populations that differ in longevity, functional capacity, and recall potential (*1*). In particular, the balance between terminally differentiated effector cells and progenitor-like memory precursors dictates the quality and durability of T cell-mediated immunity (*2*, *3*). Despite substantial progress in identifying effector and memory subsets, the mechanisms controlling CD8^+^ T cell fate decisions during priming are not yet fully understood.

Adaptive immune responses vary substantially depending on the conditions under which priming occurs. Among these, time of day has emerged as a key factor, with multiple studies reporting differences in the efficacy of vaccination and immunotherapy based on time of administration (*4*–*6*). Circadian rhythms influence multiple aspects of immune function, including lymphocyte trafficking (*7*, *8*), antibody production (*9*), and cytokine secretion (*10*, *11*). Yet, whether circadian clocks directly shape CD8^+^ T cell differentiation, and through which cellular and molecular mechanisms, remains poorly understood.

Circadian rhythms are orchestrated by a central clock in the suprachiasmatic nucleus and reinforced by cell-intrinsic clocks in most immune cell types, including T cells and dendritic cells (DCs) (*12*, *13*). DCs are central to immune priming, integrating antigen presentation with costimulatory and inflammatory cues that instruct CD8^+^ T cell differentiation (*14*). CD8^+^ T cells, in turn, translate these signals into transcriptional and metabolic programs that determine commitment to effector or memory programs. However, how clocks in these cell types coordinate memory CD8^+^ T cell differentiation is unknown.

Here, we demonstrate that the time of day of antigen encounter critically influences the differentiation trajectory of antigen-specific CD8^+^ T cells. Immunization of mice during nighttime (their active phase) promotes the generation of progenitor-like memory CD8^+^ T cells and enhances T cell-mediated antiviral protection. Mechanistically, this effect is driven by DC-intrinsic circadian clocks that regulate expression of the costimulatory ligand CD70. Together, these findings uncover a DC-mediated circadian mechanism that governs memory CD8^+^ T cell differentiation and highlight the impact of circadian timing on immune priming.

## RESULTS

### Time of antigen encounter shapes the differentiation of Spike-specific CD8^+^ T cells

**Fig. 1.**
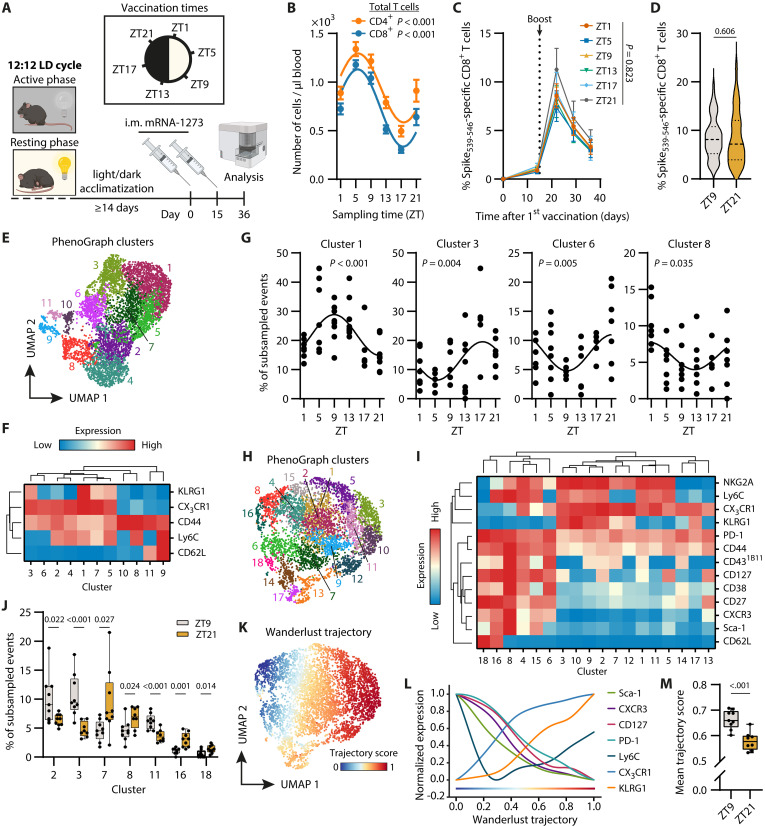
Time of antigen encounter shapes the differentiation of Spike-specific CD8^+^ T cells. (**A**) Experimental design for vaccination at different Zeitgeber times (ZTs) following acclimatization to a 12 hour:12 hour light:dark (LD) cycle. ZT0 marks light onset and ZT12 dark onset; ZT0–ZT12 corresponds to the resting phase and ZT12–ZT0 to the active phase of mice. Created in BioRender. Vleeshouwers, W. (2026) https://BioRender.com/nh4r6f4. (**B**) Circulating CD4^+^ and CD8^+^ T cell numbers in naïve mice (mean ± s.e.m.; *n* = 8 per group; representative of three independent experiments). (**C** and **D**) Frequency of Spike_539–546_-specific CD8^+^ T cells in blood over time [(C) mean ± s.e.m.; *n* = 8 per group] or on day 36 [(D) lines indicate the median and quartiles; *n* = 58 (ZT9), *n* = 60 (ZT21), pooled from six independent experiments]. (**E**) UMAP embedding of Spike_539–546_-specific cells from blood. (**F**) Corresponding hierarchically clustered heatmap showing normalized marker expression (blue, low; red, high). (**G**) Relative abundance of significantly rhythmic clusters (*n* = 8 per group). (**H** and **I**) UMAP embedding and corresponding heatmap of Spike_539–546_-specific cells from blood. Representative of three independent experiments. (**J**) Relative abundance of statistically significant clusters [*n* = 8 (ZT9), *n* = 9 (ZT21)]. (**K**) Wanderlust trajectory projected onto the UMAP in (H). (**L**) Normalized average expression of selected markers along the Wanderlust trajectory. (**M**) Mean Wanderlust trajectory scores [*n* = 8 (ZT9), *n* = 9 (ZT21)]. In (B) and (G), rhythmicity was assessed by cosine fitting; cosine waves indicates a significant fit (*P* < 0.05). Statistical analysis was performed using two-way repeated measures ANOVA in (C), the Mann-Whitney test in (D), and two-sided t-tests in [(J) and (M)]. Exact *P* values are shown on the graph.

To determine how the time of day of antigen encounter influences the CD8^+^ T cell response, we used mRNA-1273, an mRNA vaccine encoding the severe acute respiratory syndrome coronavirus 2 (SARS-CoV-2) Spike glycoprotein (*15*), as a murine vaccination model. To facilitate simultaneous vaccination and sample collection at different time points across the 24-hour period, animals were housed in light-controlled cabinets with shifted light/dark cycles (Fig. 1A). After at least 14 days of acclimatization, circulating T cell numbers exhibited 24-hour rhythmicity with a peak during the resting phase (light phase in nocturnal animals) (Fig. 1B), as described previously (*7*).

Next, we vaccinated mice at six different zeitgeber times (ZT, i.e., time notation where ZT0 is time of light onset and ZT12 is time of light offset in a 12 hour:12 hour light:dark cycle) using a prime-boost strategy (Fig. 1A). Vaccine-specific CD8^+^ T cell responses were quantified 21 days post-boost by peptide–MHC class I tetramer staining specific for an immunodominant Spike epitope (Spike_539–546_; VNFNFNGL) (*16*) (fig. S1A). Vaccination time did not affect the abundance of Spike_539–546_-specific CD8^+^ T cells (Fig. 1C and fig. S1B and C), even when assessed across independent experiments with robust sample sizes (Fig. 1D). Timing had no measurable impact on either the contraction kinetics of the T cell response or the circulating titers of Spike-specific IgG (fig. S1, D and E).

To assess the differentiation state of these cells, we performed unsupervised phenotypic analysis based on five key memory markers (fig. S1F). Dimension reduction and hierarchical clustering identified 11 distinct phenotypic clusters, showing that circulating memory cells are predominantly CD62L^−^, with only a minor CD62L^+^ central-memory subset (clusters 9 and 11) (Fig. 1, E and F). Within the CD62L^−^ clusters, phenotypic diversity was evident in the expression of CX_3_CR1 and KLRG1, markers of advanced effector differentiation (*17*, *18*), and Ly6C, a glycoprotein associated with effector cytokine production (*19*, *20*). Vaccination during the resting phase increased the frequency of Ly6C^+^CX_3_CR1^+^KLRG1^+^ cells (cluster 1), peaking at ZT9 (Fig. 1G and fig. S1G). In contrast, immunization during the late active phase enriched subsets lacking Ly6C (clusters 3 and 6) or CX_3_CR1 (cluster 8). A similar pattern was observed in the spleen, where vaccination at ZT9 (late resting phase) promoted Ly6C^+^CX_3_CR1^+^KLRG1^+^ differentiation, while vaccination at ZT21 (late active phase) favored Ly6C^−^KLRG1^−^ cells and CD62L^+^ subsets (fig. S1H). These results indicate that time of day of immunization impacts the differentiation of antigen-specific CD8^+^ T cells.

Since CX_3_CR1 and Ly6C are linked to effector function (*19*, *21*), we assessed the impact of timing of vaccination on the capacity of Spike_539–546_-specific CD8^+^ T cells to produce effector cytokines (fig. S2A). Resting phase vaccination increased interferon-gamma (IFN-γ) production compared to active phase vaccination, without affecting tumor necrosis factor (TNF) or interleukin-2 (IL-2) (fig. S2B). Together, these findings show that immunization time modulates both differentiation and effector function, with the largest differences between ZT9 (late resting phase) and ZT21 (late active phase) vaccinations.

To more comprehensively delineate the time-of-day-dependent effects on memory differentiation, we performed high-dimensional phenotyping of circulating Spike_539–546_-specific CD8^+^ T cells from mice vaccinated at ZT9 or ZT21 using a panel designed to capture the heterogeneity of antigen-specific responses (*22*). This analysis identified 18 clusters, which segregated into progenitor-like subsets with high CD127, CD27, and PD-1 expression (clusters 4, 6, 8, 15, 16, and 18), and more differentiated CX_3_CR1^+^ clusters expressing low or intermediate CD127, CD27, and PD-1 (remaining clusters) (Fig. 1, H and I and fig. S2C). Immunization time significantly altered the abundance of seven clusters (Fig. 1J and fig. S2D). ZT9 vaccination enriched three Ly6C^+^CX_3_CR1^+^PD-1^int^ clusters (2, 3, and 11), two of which expressed KLRG1 and intermediate CD127 levels (Fig. 1, I and J). In contrast, ZT21 vaccination increased the abundance of one Ly6C^−^CX_3_CR1^+^ cluster (7), phenotypically similar to the ZT9-enriched subsets, and three CXCR3^+^PD-1^hi^ clusters (8, 16, and 18). To relate these phenotypic changes to the differentiation state of Spike_539–546_-specific cells, we applied the single-cell trajectory algorithm Wanderlust (*23*) (Fig. 1K). This analysis revealed a continuum from stem-like Sca-1^+^CD127^+^ cells to effector-like CX_3_CR1^+^KLRG1^+^ cells (Fig. 1L). Along this trajectory, Sca-1, CXCR3, CD127, and PD-1 were progressively downregulated, reflecting differentiation from progenitor-like to more terminal effector states. Active phase (ZT21) vaccination yielded populations skewed toward an earlier, progenitor-like state, whereas resting phase (ZT9) vaccination favored a more differentiated, effector-like phenotype (Fig. 1M). These findings demonstrate that immunization time modulates the differentiation spectrum of antigen-specific CD8^+^ T cells, regulating the balance between progenitor-like and effector-like memory subsets.

### Time-of-day effects are maintained during late memory and throughout different tissues

**Fig. 2.**
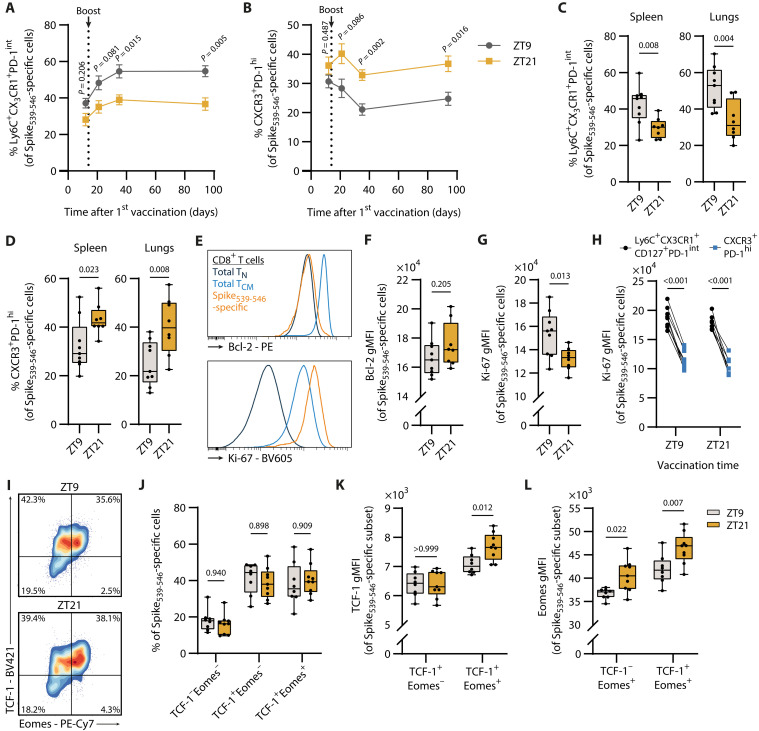
Time-of-day effects are maintained during late memory and throughout different tissues. (**A** and **B**) Frequency of Ly6C^+^CX_3_CR1^+^PD-1^int^ (A) and CXCR3^+^PD-1^hi^ (B) Spike_539–546_-specific CD8^+^ T cells in blood (mean ± s.e.m.). (**C** and **D**) Frequency of Ly6C^+^CX_3_CR1^+^PD-1^int^ (C) and CXCR3^+^PD-1^hi^ (D) Spike_539–546_-specific CD8^+^ T cells on day 95. (**E**) Representative histograms of Bcl-2 and Ki-67 expression by naïve (T_N_), central memory (T_CM_), and Spike_539–546_-specific CD8^+^ T cells in the spleen. (**F** to **H**) Geometric mean fluorescent intensity (gMFI) of Bcl-2 (F) and Ki-67 [(G) and (H)]. In (H), data points from the same mice are connected. (**I**) Representative contour plots showing TCF-1 and Eomes co-expression in Spike_539–546_-specific CD8^+^ T cells. (**J**) Frequency of TCF-1- and Eomes-expressing Spike_539–546_-specific CD8^+^ T cells from the spleen on day 95. Representative of two independent experiments. (**K** and **L**) TCF-1 and Eomes gMFI in Spike_539–546_-specific CD8^+^ T cells from the spleen. For all panels, *n* = 8 (ZT9) and *n* = 9 (ZT21). Statistical analysis was performed using two-way repeated measures ANOVA with Sidak’s post hoc test in [(A), (B), and (H)], two-way ANOVA with Sidak’s post hoc test in (J) to (L), and two-sided t-tests in [(C), (D), (F), and (G)]. Exact *P* values are shown on the graph.

To determine how immunization timing shapes long-term memory, we investigated the kinetics of time-of-day-dependent effects by focusing on Ly6C^+^CX_3_CR1^+^PD-1^int^ cells (ZT9-enriched phenotype) and CXCR3^+^PD-1^hi^ cells (ZT21-enriched phenotype). Differences between ZT9 and ZT21 were amplified by boost vaccination and persisted through both early (21 days post-boost) and late (80 days post-boost) memory phases (Fig. 2, A and B). During late memory, the enrichment of Ly6C^+^CX_3_CR1^+^PD-1^int^ cells following resting phase vaccination was also evident in both spleen and lungs (Fig. 2C), whereas active phase vaccination was associated with higher frequencies of CXCR3^+^PD-1^hi^ cells (Fig. 2D). These findings indicate that the time of immunization imprints long-lasting differences in the composition of antigen-specific CD8^+^ T cell memory across multiple organs.

Given the heterogeneous expression of CD127, which is essential for long-term memory maintenance (*24*, *25*), and KLRG1 within the ZT9-enriched population during early memory (Fig. 1J), we examined the temporal dynamics of these markers in relation to vaccination time. Prior to boosting, timing primarily influenced the CD127^−^KLRG1^+^ subset, whereas after boosting the abundance of CD127^+^ cells also became time-dependent (fig. S3). During memory maturation, the proportion of CD127^−^ cells declined and the time-of-day effect disappeared, while CD127^+^ subsets remained differentially regulated by vaccination time. These findings indicate that immunization time affects long-lasting memory subsets, supporting the conclusion that timing has durable impact on CD8^+^ T cell differentiation.

To determine how time of day influences the persistence and turnover of memory cells, we analyzed expression of the anti-apoptotic molecule Bcl-2 and the cell cycle marker Ki-67. Spike_539–546_-specific cells uniformly expressed Bcl-2 at levels comparable to naïve CD8^+^ T cells (Fig. 2E), indicating resistance to apoptosis. These cells also expressed higher levels of Ki-67 than naïve CD8^+^ T cells, suggesting ongoing homeostatic proliferation or a heightened state of readiness to divide. While Bcl-2 expression was unaffected by vaccination time (Fig. 2F), Ki-67 levels were elevated following resting phase vaccination (Fig. 2G). Subset analysis revealed higher Ki-67 expression in the ZT9-enriched Ly6C^+^CX_3_CR1^+^CD127^+^PD-1^int^ subset compared to ZT21-enriched CXCR3^+^PD-1^hi^ cells (Fig. 2H), indicating that resting phase vaccination promotes differentiation towards a homeostatically active state, while active phase vaccination favors a more quiescent memory phenotype.

Next, we asked whether these phenotypic differences were reflected at the transcriptional level. To address this, we examined expression of the transcription factors T cell factor 1 (TCF-1) and eomesodermin (Eomes), which are central regulators of memory maintenance (Fig. 2I). Most Spike_539–546_-specific cells expressed TCF-1, and this frequency was unaffected by vaccination time (Fig. 2J). However, active-phase vaccination increased the expression of both TCF-1 and Eomes within the TCF-1^+^Eomes^+^ subset (Fig. 2, K and L), indicating stronger engagement of the transcriptional program that supports memory maintenance. Collectively, these findings reveal that timing of antigen encounter shapes long-term CD8^+^ T cell memory by coordinating memory differentiation, homeostatic activity, and the transcriptional programs that preserve stem-like potential.

### Sampling time does not confound CD8^+^ T cell differentiation phenotypes

In our experimental setup (Fig. 1A), tissues were collected at the same ZT as the vaccinations. Since CD127 and PD-1 expression have been reported to exhibit diurnal oscillations (*26*, *27*), we considered the possibility that sampling time could influence our findings. To test this, we vaccinated mice at either ZT9 or ZT21 and collected tissues on day 36 either at the same ZT as the vaccinations or 12 hours later. Regardless of sampling time, vaccination at ZT9 consistently resulted in more Ly6C^+^CX_3_CR1^+^CD127^+^PD-1^int^ cells in blood, spleen, and lungs (fig. S4A). Similarly, the abundance of CXCR3^+^PD-1^hi^ cells was unaffected by collection time (fig. S4B). In conclusion, these data show that the impact of timing on Spike_539–546_-specific CD8^+^ T cell differentiation is caused by immunization time rather than sampling time.

### Time-of-immunization effects are mediated by the molecular clock of DCs

**Fig. 3.**
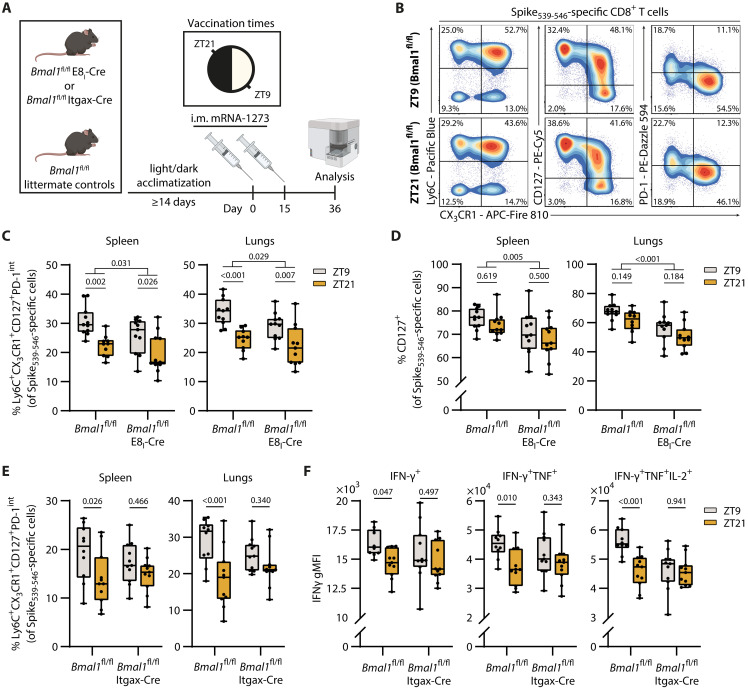
Time-of-immunization effects are mediated by the molecular clock of DCs. (**A**) Experimental design for vaccination of *Bmal1*^fl/fl^ E8_I_-Cre, *Bmal1*^fl/fl^ Itgax-Cre, and *Bmal1*^fl/fl^ littermate controls at ZT9 and ZT21. Created in BioRender. Vleeshouwers, W. (2026) https://BioRender.com/nh4r6f4. (**B**) Representative contour plots showing CX_3_CR1, Ly6C, CD127, and PD-1 expression in Spike_539–546_-specific CD8^+^ T cells from *Bmal1*^fl/fl^ mice. (**C** and **D**) Frequency of Ly6C^+^CX_3_CR1^+^CD127^+^PD-1^int^ (C) and CD127^+^ (D) Spike_539–546_-specific CD8^+^ T cells in *Bmal1*^fl/fl^ E8_I_-Cre mice and littermate controls on day 36 (*n* = 11 per group). (**E**) Frequency of Ly6C^+^CX_3_CR1^+^CD127^+^PD-1^int^ Spike_539–546_-specific CD8^+^ T cells in *Bmal1*^fl/fl^ Itgax-Cre mice and littermate controls on day 36 [*n* = 10 (*Bmal1*^fl/fl^, ZT9), *n* = 11 (other groups)]. (**F**) IFN-γ gMFI in splenic CD8^+^ T cells from *Bmal1*^fl/fl^ Itgax-Cre mice and littermate controls, shown for IFN-γ^+^ (left), IFN-γ^+^TNF^+^ (middle), and IFN-γ^+^TNF^+^IL-2^+^ (right) cells [*n* = 10 (*Bmal1*^fl/fl^, ZT9), *n* = 11 (other groups)]. Statistical analysis was performed using two-way ANOVA with Sidak’s post hoc test in (C) to (F). Exact *P* values are shown on the graph.

The induction of CD8^+^ T cell responses requires priming of T cells by antigen-presenting cells such as DCs, which provide signals that shape T cell differentiation and fate (*14*). To dissect the mechanisms underlying the time-of-day effects observed here, we assessed the contribution of the molecular clock of CD8^+^ T cells and DCs using models of conditional knock-out of the core clock gene *Bmal1* (Fig. 3A). In mice lacking BMAL1 in mature CD8^+^ T cells (*Bmal1*^fl/fl^ x E8_I_-Cre), vaccination at ZT9 still produced more Ly6C^+^CX_3_CR1^+^CD127^+^PD-1^int^ cells than vaccination at ZT21, although this effect was less pronounced than in littermate controls (Fig. 3, B and C). This indicates that the T cell-intrinsic clock contributes to time-of-day-dependent differentiation but is not the primary determinant. *Bmal1*-deficient CD8^+^ T cells also exhibited an overall reduced frequency of Ly6C^+^CX_3_CR1^+^CD127^+^PD-1^int^ cells, which could be attributed to reduced CD127 expression (Fig. 3D).

In contrast to its limited role in T cells, deletion of *Bmal1* in DCs (*Bmal1*^fl/fl^ x *Itgax*-Cre) eliminated the impact of vaccination time on CD8^+^ T cell differentiation (Fig. 3E). Moreover, *Bmal1* deficiency in DCs abolished the difference in IFN-γ production by CD8^+^ T cells between ZT9 and ZT21 vaccination (Fig. 3F). Together, these findings demonstrate that the molecular circadian clock in DCs, rather than in CD8^+^ T cells, is the principal mediator of the time-of-immunization effect on CD8^+^ T cell differentiation and function.

### Time-of-immunization effects are independent of CD28 costimulation

**Fig. 4.**
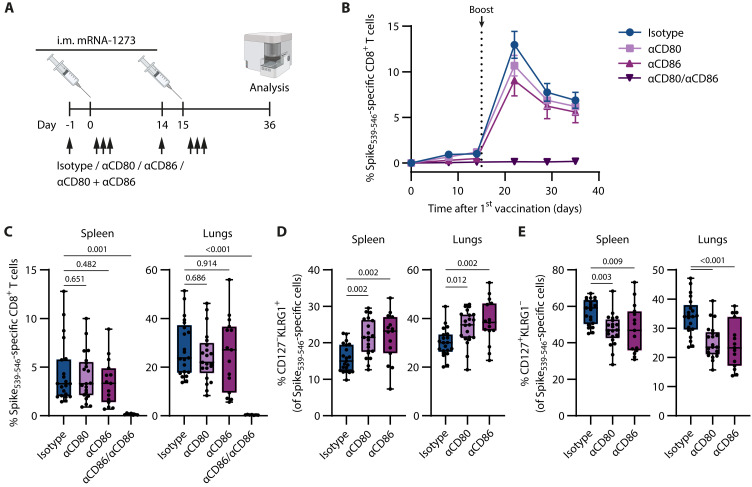
CD28 costimulation is essential for vaccine-induced CD8^+^ T cell expansion. (**A**) Experimental design for CD80 and/or CD86 blockade during response initiation. Mice were vaccinated between ZT5 and ZT10. Created in BioRender. Vleeshouwers, W. (2026) https://BioRender.com/nh4r6f4. (**B** and **C**) Frequency of Spike_539–546_-specific CD8^+^ T cells in blood over time (B) [mean ± s.e.m., *n* = 7 (αCD80/αCD86), *n* = 21 (other groups), pooled from three independent experiments], or in spleen and lungs on day 36 (C) [*n* = 7 (αCD80/αCD86), *n* = 21 (other groups)]. (**D** and **E**) Frequency of KLRG1^+^CD127^−^ (D) and KLRG1^−^CD127^+^ (E) Spike_539–546_-specific CD8^+^ T cells on day 36 [*n* = 21 (isotype and αCD80), *n* = 15 (αCD86), pooled from three independent experiments]. Statistical analysis was performed using one-way ANOVA with Dunnett’s post hoc test in (C) to (E). Exact *P* values are shown on the graph.

We next sought to identify the DC-derived signals mediating time-of-day-dependent effects on T cells. Previous work has shown that circadian variation in anti-tumor CD8^+^ T cell responses is driven by circadian rhythmicity in CD80 expression on DCs (*28*). Costimulatory ligands like CD80 and CD86, typically expressed on DCs (*29*), provide key signals that shape T cell immunity. However, their role in shaping responses to mRNA-1273 remains unclear. To address this, we first assessed the importance of CD80- and CD86-mediated costimulatory signaling through CD28 on T cells by immunizing mice treated with blocking antibodies against these ligands (Fig. 4A). Disruption of both CD80- and CD86-mediated costimulation fully abrogated the Spike_539–546_-specific CD8^+^ T cells response (Fig. 4, B and C), demonstrating that CD28 engagement is crucial for vaccine-induced expansion. In contrast, inhibition of either CD80 or CD86 alone did not substantially alter the magnitude of the response, indicating that these ligands exhibit functional redundancy in the response to mRNA-1273. Phenotypic analysis revealed that blockade of CD80 or CD86 favored the differentiation towards an effector-like CD127^−^KLRG1^+^ phenotype, which was accompanied by a relative reduction in CD127^+^KLRG1^−^ cells (Fig. 4, D and E). This finding was corroborated by hierarchical clustering analysis (fig. S5). Together, these findings demonstrate that CD28 signaling is indispensable for the expansion of Spike_539–546_-specific CD8^+^ T cells and that selective disruption of CD80- or CD86-mediated signaling promotes differentiation towards a more effector-like state.

**Fig. 5.**
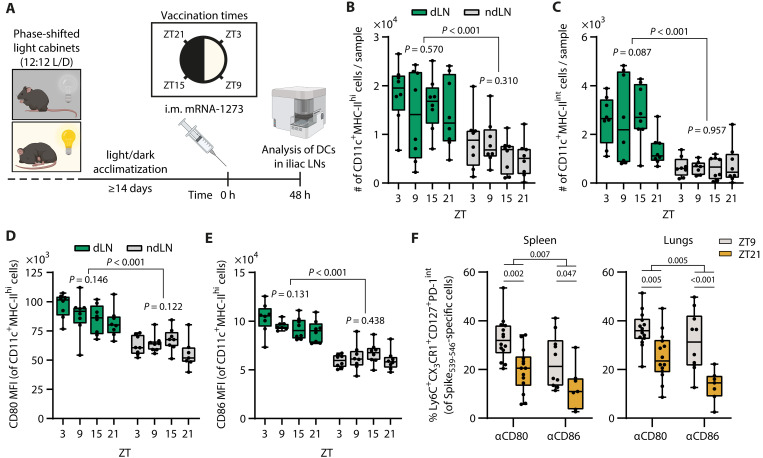
Time-of-immunization effects are independent of CD28 costimulation. (**A**) Experimental design for analysis of DCs in iliac LNs following vaccination at different ZT. Created in BioRender. Vleeshouwers, W. (2026) https://BioRender.com/nh4r6f4. (**B** and **C**) Number of CD11c^+^MHC-II^hi^ (B) and CD11c^+^MHC-II^int^ (C) DCs in the draining LN (dLN) and non-draining LN (ndLN) 48 hours after vaccination (*n* = 8 per group). (**D** and **E**) Mean expression of CD80 (D) and CD86 (E) on DCs 48 hours after vaccination (*n* = 8 per group). (**F**) Frequency of Ly6C^+^CX_3_CR1^+^CD127^+^PD-1^int^ Spike_539–546_-specific CD8^+^ T cells on day 36 in mice treated with αCD80 or αCD86 [*n* = 14 (αCD80), *n* = 10 (αCD86, ZT9), *n* = 7 (αCD86, ZT21); pooled from two independent experiments]. Statistical analysis was performed using one-way ANOVA for time effects in (B) to (E), two-way repeated-measures ANOVA for organ effects in (B) to (E), and two-way ANOVA with Sidak’s post hoc test in (F). Exact *P* values are shown on the graph.

Next, we examined whether CD80 and CD86 contribute to the effect of timing on CD8^+^ T cell differentiation. To this end, we quantified surface expression of CD80 and CD86 on DCs in the vaccine-draining lymph node (dLN) 48 hours after immunization (Fig. 5A). Consistent with a prior report (*30*), we confirmed that the ipsilateral iliac lymph node serves as the primary drainage site after intramuscular injection (fig. S6A). The contralateral iliac lymph node was used as non-draining control (ndLN).

Within these lymph nodes, we identified CD11c^+^MHC-II^hi^ DCs expressing either CD103 or CD11b, as well as CD11c^+^MHC-II^int^ DCs expressing CD8α or CD11b (fig. S6B). Vaccination increased total DC numbers in the dLN (Fig. 5, B and C). Although vaccination time did not affect the abundance of MHC-II^hi^ DCs, MHC-II^int^ DCs were slightly reduced following vaccination at ZT21. As expected, DCs in the dLN expressed higher levels of the maturation markers CD80 and CD86 than those in the ndLN (Fig. 5, D and E, and fig. S6, C and D). However, the expression of both CD80 and CD86 was not significantly affected by vaccination time. Similarly, CD28 levels on CD8^+^ T cells were not impacted by vaccination time (fig. S6E). These observations imply that the time-dependent effects in our model are not driven by CD28-mediated costimulation. To corroborate this, we vaccinated mice at ZT9 or ZT21 while blocking either CD80 or CD86. Even in the absence of either CD80- or CD86-mediated CD28 activation, ZT9 vaccination continued to elicit a more effector-like CD8^+^ T cell differentiation phenotype marked by a higher frequency of Ly6C^+^CX_3_CR1^+^CD127^+^PD-1^int^ cells (Fig. 5F). Together, these findings indicate that CD28-mediated costimulation does not play a substantial role in coordinating time-of-day-dependent differentiation following mRNA vaccination.

### Time-of-day-dependent CD70 induction shapes T cell differentiation

**Fig. 6.**
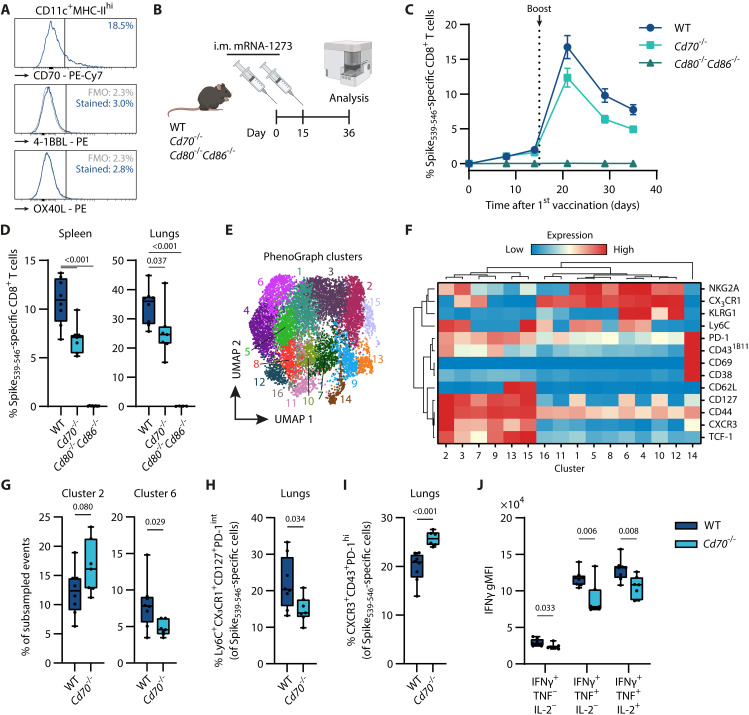
CD27 costimulation shapes CD8^+^ T cell differentiation and function. (**A**) Representative histograms showing CD70, 4-1BBL, and OX40L expression on CD11c^+^MHC-II^hi^ cells in the dLN 48 hours after vaccination. (**B**) Experimental design for vaccination of *Cd70*^−/−^ and *Cd80*^−/−^*Cd86*^−/−^ mice with mRNA-1273 (between ZT5 and ZT10). Created in BioRender. Vleeshouwers, W. (2026) https://BioRender.com/nh4r6f4. (**C** and **D**) Frequency of Spike_539–546_-specific CD8^+^ T cells in blood over time (C) (mean ± s.e.m.), or in spleen and lungs on day 36 (D) [*n* = 8 (WT), *n* = 7 (*Cd70*^−/−^), *n* = 4 (*Cd80*^−/-^*Cd86*^−/−^)]. (**E**) UMAP embedding of Spike_539–546_-specific cells from spleens of WT and *Cd70*^−/−^ mice. (**F**) Corresponding hierarchically clustered heatmap showing normalized marker expression (blue, low; red, high). (**G**) Relative abundance of PhenoGraph clusters [*n* = 8 (WT), *n* = 7 (*Cd70*^−/−^)]. (**H** and **I**) Frequency of Ly6C^+^CX_3_CR1^+^CD127^+^PD-1^int^ (H) and CXCR3^+^CD43^+^PD-1^hi^ (I) Spike_539–546_-specific CD8^+^ T cells in the lungs on day 36 [*n* = 8 (WT), *n* = 6 (*Cd70*^−/−^)]. (**J**) IFN-γ gMFI in CD8^+^ T cells from WT and *Cd70*^−/−^ mice [*n* = 8 (WT), *n* = 7 (*Cd70*^−/−^)]. Statistical analysis was performed using one-way ANOVA with Dunnett’s post hoc test in (D) and two-sided t-tests in (G) to (J). Exact *P* values are shown on the graph.

To determine whether additional DC-derived costimulatory signals mediate time-of-day-dependent CD8^+^ T cell differentiation, we examined receptor–ligand pairs of the TNF receptor superfamily known to regulate CD8^+^ T cell responses, including CD27–CD70, 4-1BB–4-1BBL, and OX40–OX40L (*31*). To evaluate their involvement in responses to mRNA-1273 vaccination, we quantified the expression of CD70, 4-1BBL, and OX40L on DCs in the dLN. While 4-1BBL and OX40L were undetectable, CD70 was expressed on a subset of DCs (Fig. 6A), suggesting that CD70-mediated costimulation may modulate CD8^+^ T cell priming in this setting.

To test this, we vaccinated *Cd70*-deficient mice with mRNA-1273 and analyzed the Spike_539–546_-specific CD8^+^ T cell response, using *Cd80*^−/−^*Cd86*^−/−^ mice as negative controls (Fig. 6B). While the negative controls failed to mount detectable responses, *Cd70* deficiency modestly reduced the magnitude of the response compared to wild-type mice (Fig. 6, C and D). To assess how CD70 regulates CD8^+^ T cell differentiation, we then performed dimensionality reduction and unsupervised clustering of Spike_539–546_-specific CD8^+^ T cells from the spleen (Fig. 6, E and F, and and fig. S7A). Loss of CD70–CD27 signaling altered memory differentiation, increasing the proportion of progenitor-like CXCR3^+^CD43^+^PD-1^hi^ cells (cluster 2) and reducing effector-like CX_3_CR1^+^Ly6C^+^PD-1^int^CD127^int^ cells (cluster 6) (Fig. 6G and fig. S7B). Similar changes were observed in the lungs (Fig. 6, H and I). Notably, cluster 2 and 6 closely resemble the ZT21- and ZT9-enriched differentiation phenotypes, respectively, indicating that CD70 signaling modulates CD8^+^ T cell differentiation in a manner similar to time of immunization.

Because a progenitor-like memory phenotype is associated with lower IFN-γ production (*21*), we next assessed cytokine output in *Cd70*^−/−^ mice. Consistent with the differentiation pattern, IFN-γ production was reduced in absence of CD27 signaling (Fig. 6J). Collectively, these results demonstrate that CD70–CD27 interactions regulate the expansion, differentiation, and effector function of vaccine-specific CD8^+^ T cells.

**Fig. 7.**
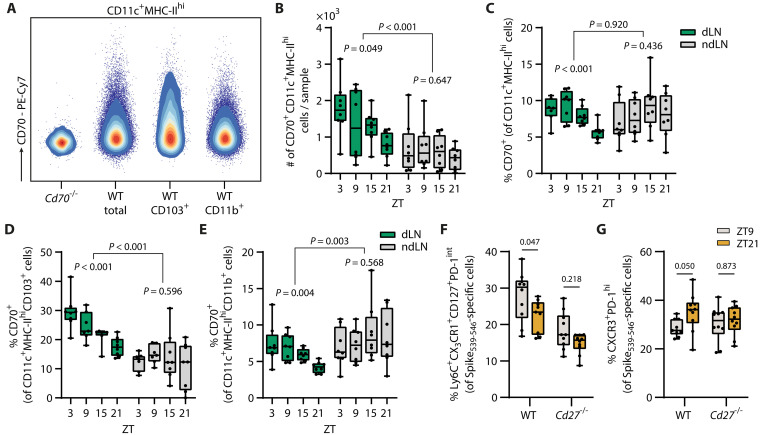
Time-of-day-dependent induction of CD70 shapes T cell differentiation. (**A**) Representative contour plots showing CD70 expression on CD11c^+^MHC-II^hi^ DCs. (**B**) Number of CD70^+^CD11c^+^MHC-II^hi^ cells in dLN and ndLN 48 hours after vaccination (*n* = 8 per group). (**C** to **E**) Frequency of CD70^+^ cells among CD11c^+^MHC-II^hi^ (C), CD11c^+^MHC-II^hi^CD103^+^ (D), CD11c^+^MHC-II^hi^CD11b^+^ (E) cells in dLN and ndLN 48 hours after vaccination (*n* = 8 per group). (**F** and **G**) Frequency of Ly6C^+^CX_3_CR1^+^CD127^+^PD-1^int^ (F) and CXCR3^+^PD-1^hi^ (G) Spike_539–546_-specific CD8^+^ T cells in the spleen on day 36 [*n* = 9 (WT, ZT21), *n* = 10 (other groups)]. Statistical analysis was performed using one-way ANOVA for time effects in (B) to (E), two-way repeated-measures ANOVA for organ effects in (B) to (E), and two-way ANOVA with Sidak’s post hoc test in [(F) and (G)]. Exact *P* values are shown on the graph.

Given that CD70-mediated costimulation influences Spike-specific CD8^+^ T cell differentiation, we next asked whether time of antigen encounter alters CD70 expression on DCs. In the dLN of mice vaccinated with mRNA-1273, CD70 expression was restricted to a subset of MHC-II^hi^ DCs, with the highest levels observed in the MHC-II^hi^CD103^+^ population (Fig. 7A and fig. S8A). Vaccination increased the number of CD70^+^ DCs, and this effect was most pronounced in mice immunized during the resting phase (Fig. 7B). Consistent with these absolute counts, the frequency of CD70^+^ DCs also peaked after resting phase vaccination, both overall and within the CD11b^+^ and CD103^+^ subsets analyzed separately (Fig. 7, C to E, and fig. S8, B and C). Immunization time no longer affected CD70 expression in mice with *Bmal1*-deficient DCs, demonstrating that this effect is regulated by the DC-intrinsic circadian clock (fig. S8D). This time-of-vaccination effect was not observed in the ndLN or in the lymph nodes of unvaccinated mice (Fig. 7, B to E, and fig. S8E), indicating its specificity for DCs responding to immunization. In contrast to CD70, CD27 expression on CD8^+^ T cells was unaffected by vaccination time (fig. S8F). Thus, resting phase vaccination results in the greatest induction of CD70^+^ DCs in the dLN.

To determine the impact of this time-of-day-dependent induction of CD70^+^ DCs on CD8^+^ T cell memory, we assessed Spike-specific responses in *Cd27*^−/−^ animals vaccinated at ZT9 or ZT21. Consistent with our observations in *Cd70*-deficient mice (Fig. 6D), loss of CD27 signaling reduced the magnitude of the antigen-specific response regardless of vaccination time (fig. S8G). In wild-type mice, resting phase vaccination increased the frequency of effector-like Ly6C^+^CX_3_CR1^+^CD127^+^PD-1^int^ cells and reduced the frequency of progenitor-like CXCR3^+^PD-1^hi^ cells relative to active phase vaccination (Fig. 7, F and G), as observed previously. This time-dependent effect was lost in *Cd27*^−/−^ mice, indicating that CD70–CD27 costimulatory signaling is required for the time-of-day-dependent regulation of CD8^+^ T cell differentiation.

### Active phase immunization enhances T cell-mediated antiviral protection

**Fig. 8.**
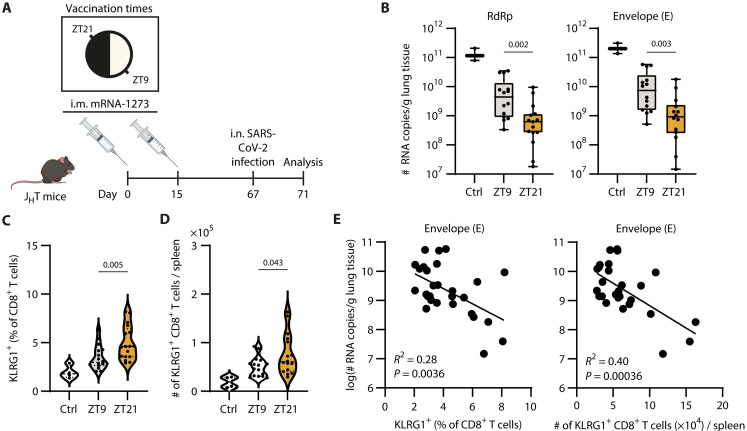
Active phase immunization enhances T cell-mediated antiviral protection. (**A**) Experimental design for vaccination and SARS-CoV-2 challenge of J_H_T mice. Created in BioRender. Vleeshouwers, W. (2026) https://BioRender.com/nh4r6f4. (**B**) qPCR analysis of SARS-CoV-2 RNA-dependent RNA polymerase (RdRp) and envelope (E) protein RNA in the lungs [*n* = 3 (control), *n* = 14 (other groups)]. (**C** and **D**) Frequency (C) and number (D) of KLRG1^+^ CD8^+^ T cells in the spleen [*n* = 4 (control), *n* = 16 (other groups)]. (**E**) Correlation between frequency (left) or number (right) of KLRG1^+^ CD8^+^ T cells in the spleen and viral E protein RNA in the lungs (*n* = 28). Statistical analysis was performed using the Mann-Whitney test in (B) to (D) and linear regression with an F test in (E). Exact *P* values are shown on the graph.

To specifically assess how immunization time influences CD8^+^ T cell-mediated antiviral protection independently of vaccine-induced neutralizing antibodies, B cell-deficient J_H_T mice were vaccinated at ZT9 or ZT21 and subsequently challenged with SARS-CoV-2 (Fig. 8A). Four days post-challenge, mice vaccinated at ZT21 exhibited the lowest viral RNA in the lungs (Fig. 8B). Only modest bodyweight loss was observed following challenge, with no differences between experimental groups (fig. S9A). This is consistent with the relatively limited clinical disease severity of this model compared to more severe SARS-CoV-2 infection models, such as K18-hACE2 transgenic mice (*32*). To evaluate CD8^+^ T cell expansion during infection, we quantified KLRG1^+^ CD8^+^ T cells in the spleen as a surrogate for effector cell expansion. Animals vaccinated at ZT21 exhibited higher frequencies and numbers of KLRG1^+^ CD8^+^ T cells (Fig. 8, C and D), which inversely correlated with lung viral RNA levels (Fig. 8E and fig. S9, B and C), indicating that increased effector CD8^+^ T cell expansion improved viral control. Together, these results demonstrate that active phase vaccination enhances T cell-mediated protection against SARS-CoV-2 infection.

## DISCUSSION

Here, we investigated how circadian rhythms shape the quality of antigen-specific CD8^+^ T cell responses. Unsupervised clustering revealed that antigen encounter during the resting phase favored an effector-memory phenotype defined by Ly6C, intermediate PD-1, and CX_3_CR1 expression. In line with the established link between CX_3_CR1 and effector differentiation (*21*), resting-phase immunization elicited a response characterized by higher IFN-γ and Ki-67 levels, whereas active-phase immunization induced a more quiescent, stem-like state. Subsets enriched following both active- and resting-phase vaccination expressed CD127 as memory differentiation progressed, indicating long-lived potential despite divergent differentiation trajectories. Importantly, these time-of-immunization effects persisted up to 80 days post-boost, aligning with prior studies showing that timing of initial immune activation can imprint long-lasting effects on T cell fate (*12*, *27*, *33*).

Functionally, active-phase immunization enhanced effector expansion upon viral challenge, resulting in superior control of acute SARS-CoV-2 infection. Thus, differentiation toward a stem-like memory phenotype, as induced by active-phase vaccination, conferred a protective advantage in this context. However, the optimal timing of immunization may depend on the desired immune outcome. While a stem-like program may favor recall and durability, (therapeutic) settings such as cancer may benefit from robust effector differentiation.

Our vaccine efficacy experiments were performed in B cell-deficient J_H_T mice to evaluate the contribution of T cell immunity to antiviral protection in the absence of vaccine-induced neutralizing antibodies. While this approach enabled assessment of time-of-vaccination-dependent effects on T cell-mediated viral control, it remains to be determined how timing of mRNA vaccination influences protection in fully immunocompetent settings. In addition, the relatively limited disease severity in this model may reduce the ability to detect differences in clinical parameters such as weight loss or survival. Future studies using infection models with greater baseline disease severity, together with conditional clock-deficient and costimulation-deficient models, will therefore be important to further define how circadian timing shapes vaccine efficacy and clinical outcome.

Mechanistically, DC-intrinsic circadian clocks proved essential for the observed time-of-day variation in CD8^+^ T cell responses, consistent with prior studies using ovalbumin-based immunization models (*28*, *34*). Although T cell-intrinsic clocks have also been implicated in circadian regulation of adaptive immunity (*12*, *28*, *33*), CD8^+^ T cell-specific deletion of *Bmal1* only modestly attenuated time-dependent effects in our system. These results suggest that the relative contribution of DCs and T cells to circadian regulation of CD8^+^ T cell differentiation may vary depending on antigen properties, adjuvant use, and antigen processing requirements (*34*). Importantly, the E8_I_-Cre model used here selectively disrupts circadian clock function in CD8^+^ T cells while preserving circadian regulation in CD4^+^ T cells, thereby avoiding potential confounding effects of altered CD4^+^ T cell help, which can enhance CD8^+^ T cell priming by stimulating DC function (*35*). This distinction may explain discrepancies with studies using *Cd4*-Cre models (*28*, *33*), where both CD4^+^ and CD8^+^ T cell circadian clocks are disrupted.

Although CD80 expression has been reported to follow circadian rhythms (*28*), our data point to CD70 as the primary driver of time-of-day effects in our model. Consistent with this role, disruption of CD27 signaling limited Spike-specific memory differentiation and reduced IFN-γ production. While *Cd70* or *Cd27* deficiency also reduced the overall magnitude of the response, time of vaccination did not affect the frequency of Spike-specific CD8^+^ T cells. This may reflect residual CD70 signaling following vaccination at ZT21, which could be sufficient to sustain expansion despite impaired effector differentiation.

While CD80- or CD86-mediated signaling did not account for the observed time-of-day effects on CD8^+^ T cell differentiation, our data show that CD28 ligation is nonetheless required for the expansion of Spike-specific CD8^+^ T cells. This requirement contrasts with highly inflammatory settings such as lymphocytic choriomeningitis virus (LCMV) infection, in which alternative costimulatory pathways can compensate for impaired CD28 signaling (*36*). In comparatively low-inflammatory contexts, CD28-dependent costimulation may therefore become indispensable for effective T cell priming. CD80 and CD86 appeared largely redundant in our system, as blockade of either molecule alone had minimal impact on T cell expansion. Such redundancy among costimulatory pathways is well documented and varies depending on the immunization context and pathogen encountered (*37*).

Beyond costimulation, other circadian-regulated processes like antigen processing and DC migration may contribute to time-dependent effects (*34*, *38*). Although we did not detect differences in total DC abundance within the dLN, circadian rhythms in migration dynamics could have been obscured by inflammation-induced DC accumulation at the time of sampling. It is likely that multiple circadian-regulated processes converge to shape the immune response (*33*); our study extends this framework by identifying a mechanism through which time of day influences T cell immunity.

We employed mRNA-based immunization as a clinically relevant platform to study circadian regulation of immune priming. SARS-CoV-2 mRNA vaccines elicit robust CD8^+^ T cell responses with broad cross-reactivity to emerging viral variants (*39*, *40*). Beyond COVID-19, the flexibility and scalability of mRNA platforms have accelerated their application for other infectious diseases and personalized cancer immunotherapy (*41*, *42*). Despite these successes, substantial interindividual variability in T cell responses remains (*43*), underscoring the importance of better understanding how factors such as circadian timing influence memory CD8^+^ T cell differentiation and function. Such interindividual variability is likely influenced by multiple biological factors beyond circadian timing alone, including sex-dependent differences in T cell activation (*44*). As this study was performed exclusively in female mice to maintain experimental consistency, future studies including both sexes will be required to determine whether sex-dependent differences influence circadian regulation of T cell immunity.

In humans, the influence of circadian cues on T cell immunity remains poorly defined. Studies examining timing of vaccination report variable results across populations, vaccine platforms, and study designs (*5*). In the context of mRNA vaccination, a large-scale retrospective analysis associated morning SARS-CoV-2 vaccination with enhanced protection (*45*), whereas most studies measuring antibody titers observed no or only transient time-of-day effects (*5*). Importantly, none directly evaluated the impact of circadian timing on cellular immune responses or memory CD8^+^ T cell differentiation. As immunotherapeutic strategies increasingly seek to elicit durable T cell-mediated immunity across infectious diseases, vaccination, and cancer, defining how the time of day shapes T cell fate decisions will be of growing importance. Our findings raise the possibility that aligning immune priming with circadian time influences the quality of memory CD8^+^ T cell responses in humans and provide a rationale for future studies explicitly designed to evaluate time-of-day effects on cellular immunity across diverse disease contexts.

## MATERIALS AND METHODS

### Study design

This study was designed to determine how time of antigen encounter influences CD8^+^ T cell differentiation and memory formation. Experiments were performed in mice immunized with SARS-CoV-2 mRNA vaccines at defined ZTs, and antigen-specific CD8^+^ T cell responses were analyzed by spectral flow cytometry. To determine the role of cell-intrinsic circadian clocks, T cell- and DC-specific *Bmal1*-deficient mice were used. Costimulation-deficient mice were used to assess the role of costimulatory signals in mediating time-of-day effects. All experiments included age- and sex-matched controls, using littermates where possible. Mice were randomly assigned to experimental groups. Sample sizes were based on power analysis of pilot data or prior experience with comparable vaccination models. Investigators were not blinded to group allocation for time-of-day interventions, but were blinded to genotype and treatment where possible.

### Mice

Wild-type C57BL/6JRj mice were purchased from Janvier Labs. *Bmal1*^fl/fl^ (B6.129S4(Cg)-*Bmal1^tm1Weit^*/J, RRID:IMSR_JAX:007668) (*46*) and J_H_T (B6.129P2-*Igh-J^tm1Cgn^*/J, RRID:IMSR_JAX:002438) (*47*) mice were obtained from The Jackson Laboratory and bred in-house. *Cd70*^−/−^ mice (*48*), *Cd80*^−/−^*Cd86*^−/−^ (B6.129S4-Cd80^tm1Shr^ Cd86^tm2Shr^/J, RRID:IMSR_JAX:003610) (*49*) mice, and *Cd27*^−/−^ mice (*50*) were bred in-house on a C57BL/6 background. *Bmal1*^fl/fl^ x E8_I_-Cre and *Bmal1*^fl/fl^ x *Itgax*-Cre mice were generated by crossing *Bmal1*^fl/fl^ mice with E8_I_-Cre mice (C57BL/6-Tg(Cd8a-cre)1Itan/J, RRID:IMSR_JAX:008766) (*51*) and *Itgax*-Cre mice (B6.Cg-Tg(Itgax-cre)1-1Reiz/J, RRID:IMSR_JAX:008068) (*52*), respectively. Genotyping was performed by Transnetyx. All mice were female and 8–16 weeks of age at the start of the experiment. Animals were housed under specific-pathogen-free conditions at the animal facility of the Leiden University Medical Center (LUMC). Mice were housed at 20–22°C and 35–65% humidity under a 12 hour:12 hour light:dark schedule with ad libitum access to food and water. To simultaneously treat animals at different timepoints, mice were housed in light-tight cabinets for at least 14 days prior to the start of the experiment. All procedures performed during the dark phase were conducted under dim red light. SARS-CoV-2 infection experiments were performed in a class 3 biological safety cabinet in the BSL3 unit of the LUMC Central Animal Facility (DM3 unit), for which mice were housed in individually ventilated isolator cages (IsoCage Biocontainment System, Tecniplast) under a light/dark cycle of 6:30 h–7:00 h sunrise, 07:00 h–18:00 h daytime and 18:00 h–18:30 h sunset. All animal experiments were approved by the national Central Animal Testing Committee (permit number AVD1160020186804 and AVD11600202417987), the Animal Tests Committee, and the Animal Welfare Body of the LUMC, and performed according to the recommendations and guidelines set by LUMC and the Dutch Experiments on Animals Act. Animal experiments were conducted in accordance with ARRIVE guidelines.

### Vaccinations

Clinical-grade mRNA-1273 (Moderna, Inc.) was stored at −70°C. Mice were immunized intramuscularly with 1 μg of mRNA-1273 in 50 μl PBS, administered into the right quadriceps. Vaccination times are indicated as Zeitgeber time (ZT), with ZT0–ZT12 referring to the light phase and ZT12–ZT24/ZT0 to the dark phase. Booster immunizations were given on day 14 or 15 at the same ZT as the primary dose. If not specified, animals were vaccinated between ZT5 and ZT10.

### In vivo antibody treatments

For CD80 or CD86 blockade, 200 μg of antibodies targeting CD80 (clone 16-10A1, BioXcell, Cat# BE0024, RRID:AB_1107676) or CD86 (clone GL1, BioXcell, Cat# BE0025, RRID:AB_1107678) or isotype control (BioXcell, Cat# BE0089, RRID:AB_1107769, and Cat# BE0091, RRID:AB_1107773) were injected intraperitoneally 1 day before each vaccination and repeated three more times every 2–3 days.

### SARS-CoV-2 infection

Mice were challenged intranasally (i.n.) under isoflurane anesthesia with 10^5^ PFU SARS-CoV-2 Beta variant B.1.351 (hCoV-19/Belgium/rega-1920/2021, EPI_ISL_896474; kindly provided by Piet Maes, KU Leuven) in 50 μl DMEM. Virus inoculations were performed on day 67 between ZT3 and ZT5. Infected mice were monitored daily for weight loss and clinical signs. At 4 days post-infection, mice were euthanized with an overdose of sodium pentobarbital (Euthasol, 200 mg/kg, injected intraperitoneally under isoflurane anesthesia) and lungs and spleens were harvested for virological (RT-qPCR) and immunological analyses.

### RNA isolation and RT-qPCR

The quantification of SARS-CoV-2 viral RNA was performed using lung homogenates of a single lung lysed with TriPure isolation reagent (Roche Applied Science) in gentleMACS M Tubes (Miltenyi Biotec) according to the manufacturer’s instructions. RNA was extracted by the addition of chloroform and liquid phases were separated by centrifugation, after which RNA was precipitated from the aqueous phase using isopropanol. SARS-CoV-2 viral copy numbers were determined by RT-qPCR using the TaqMan Fast Virus 1-Step Master Mix (Applied Biosystems) on a CFX384 Touch Real-Time PCR Detection System (BioRad). The sub-genomic mRNA PCR primers and probes against the Envelope (E) gene and the genomic RNA-dependent RNA polymerase (RdRp) gene were modified based on previously described primer and probe sets (*53*). The sub-genomic mRNA PCR primers (forward-GTGARATGGTCATGTGTGGCGG-RdRp_Sarbeco_F) and reverse-CARATGTTAAAAACACTATTAGCATA-RdRp_Sarbeco_R) and probe (FAM-CAGGTGGAACCTCATCAGGAGATGC-BHQ1-RdRp_Sarbeco_Probe) and the genomic RNA PCR primers (forward-ACAGGTACGTTAATAGTTAATAGCGT-E_Sarbeco_F and reverse-ATATTGCAGCAGTACGCACACA-E_Sarbeco_R) and probe (TexRed-ACACTAGCCATCCTTACTGCGCTTCG-BHQ2) were used. RNA samples were analyzed in triplicate. A standard curve was obtained using an in vitro transcript derived from a synthetic plasmid that contained all PCR targets. Data were normalized to sample weight. Samples with undetectable viral RNA were excluded from the analysis.

### Antigen-binding ELISA

Spike-specific antibody titers were determined by ELISA as described previously (*32*). In short, MaxiSorp ELISA plates (Nunc) were coated with Spike S1 + S2 ECD-His recombinant protein (SinoBiologicals) in ELISA coating buffer (Biolegend) and plates were blocked with 1% bovine serum albumin (Merck) in PBS with 0.05% Tween (Sigma). Plates were then incubated with serial dilutions of mouse sera and Goat Anti-Mouse IgG(H + L)-horse radish peroxidase (HRP) (SouthernBiotech, Cat# 1036–05, RRID:AB_2794348). To develop the plates, 3,3′,5,5′-tetramethylbenzidine (Sigma) was added and later quenched by the addition of 1 M H_2_SO_4_. Absorbance was acquired on a microplate reader (Model 680, Bio-Rad) at 450 nm.

### Tissue digestion and single cell preparation

Peripheral blood was collected from the tail vein. Single-cell suspensions of spleen and lymph nodes were obtained by passing the tissue over a 70 μm cell strainer. Whenever DCs were analyzed, spleens and lymph nodes were first injected with IMDM (Gibco) supplemented with 1 mg/ml Collagenase type D (Roche) and 20 μg/ml DNase I Type IV (Sigma) and incubated for 30 min at 37°C. Erythrocytes were depleted using a hypotonic ammonium chloride lysis buffer. Lung samples were cut into 1 mm^2^ pieces using surgical knives and the tissue was incubated in IMDM supplemented with 1 mg/ml Collagenase type D and 20 μg/ml DNase I Type IV for 30 min at 37°C. Lung tissue was dissociated into single-cell suspensions by passing over a 70 μm cell strainer and lymphocytes were isolated by density centrifugation on a 40%/70% Percoll (Cytiva) gradient.

### Flow cytometry

Fc receptors were blocked using TruStain FcX Plus (Biolegend, Cat# 156604, RRID:AB_2783138) and cells were incubated with fluorophore-conjugated antibodies and tetramers for 30 min at 4°C. Cells were washed with PBS supplemented with 1% FCS and 0.2% sodium azide. All antibodies used are listed in Supplementary Table S1. Fixable Zombie Aqua (Biolegend) or Zombie NIR (Biolegend) staining was used to exclude dead cells. Spike_539–546_ (VNFNFNGL)-specific CD8^+^ T cells were detected using H-2D^b^ tetramers generated at the LUMC peptide facility. When staining with tetramers, cells were pre-incubated with 50 nM Dasatinib (Sigma) for 15 min at 37°C. To stain for transcription factors or other nuclear proteins, the Foxp3/Transcription Factor Staining Buffer Set (eBioscience) was used according to the manufacturer’s instructions. When using biotin-conjugated antibodies, cells were stained with streptavidin-PE (eBioscience). To determine absolute cell counts, Precision Count Beads (Biolegend) were added to the samples at the start of the staining procedure.

Acquisition was performed on a 3- or 5-laser Cytek Aurora spectral analyzer with SpectroFlo software (v3) and data were analyzed in OMIQ (Dotmatics). Files used for dimensionality reduction were first cleaned with FlowAI using default parameters and subsampled to 6013 (Fig. 1E), 5003 (Fig. 1H), 2709 (fig. S5), or 9606 (Fig. 6) CD3^+^CD8^+^CD4^−^ Spike_539–546_-specific cells. Dimensionality reduction was performed by Uniform Manifold Approximation and Projection (UMAP) using default parameters and cluster identification was performed using PhenoGraph with the Louvain method. Clustered heatmaps of concatenated files were generated using normalized median marker expression of each cluster in Morpheus software (https://software.broadinstitute.org/morpheus/). Hierarchical clustering was performed using Euclidian distance and the average-linkage method for both rows and columns. Wanderlust trajectory analysis was performed in OMIQ using default parameters, with CD62L^+^ cells defined as the starting population.

### Intracellular cytokine staining

Single cell suspensions were stimulated with 2 μg/ml short Spike_539–546_ peptide (VNFNFNGL; generated by LUMC peptide facility) for 5 hours in IMDM supplemented with heat-inactivated FCS (Bodinco BV), 2 mM L-Glutamine (Gibco), 50 U/mL Penicillin–Streptomycin (Gibco), 25 μM β-mercaptoethanol (Sigma), and Brefeldin A (Biolegend). After staining of surface markers, cells were fixed with 4% PFA (Biolegend), permeabilized with a saponin-based wash buffer (Biolegend) and stained for cytokines by incubating with fluorophore-conjugated antibodies in permeabilization buffer for 60 min at room temperature.

### Statistical analyses

Statistical analyses were performed using GraphPad Prism (version 10). 24-hour rhythms with more than four time points were tested by cosinor analysis with a fixed 24-hour period {y = M + A*cos[(2*π*t/24) + Ps], where A is the amplitude, t is the time point (independent variable), Ps is the acrophase (time of peak), and M is the mesor (rhythm-adjusted mean)}. Statistical significance was determined by comparing the cosine fit to a horizontal line (slope = 0), representing the null hypothesis of no 24-hour rhythmicity, with a sum-of-squares F-test. Twenty-four hour variation with four time points were tested by one-way ANOVA. Unpaired t-tests (2 groups) and one- or two-way ANOVA (>2 groups) were used for other data. Whenever two-way ANOVA was used, a full model with interaction between model terms was fitted. All performed pairwise comparisons have been shown. Outliers were excluded using Grubbs’s test (*P* = 0.05). Samples with fewer than 40 events of interest were excluded from the analysis to ensure statistical robustness. All replicates represent biological replicates (individual mice), unless stated otherwise. *P* values are two-sided and *P* < 0.05 was considered statistically significant.
