# Supplementary material for: Dendritic cell circadian clocks shape memory CD8+ T cell differentiation
Source: Sci Adv. 2026 Jul 31;12(31):eaeh3719. doi: 10.1126/sciadv.aeh3719 (PMC13426428; doi:10.1126/sciadv.aeh3719)
Supplement: Supplementary file 1 — Figs. S1 to S9 Table S1 [file sciadv.aeh3719_sm.pdf]

Science Advances
AAAS

# Supplementary Materials for

## Dendritic cell circadian clocks shape memory $CD8^+$ T cell differentiation

Ward Vleeshouwers *et al.*

Corresponding author: Laura Kervezee, l.kervezee@lumc.nl; Ramon Arens, r.arens@lumc.nl

*Sci. Adv.* **12**, eaeh3719 (2026)
DOI: 10.1126/sciadv.aeh3719

**This PDF file includes:**

Figs. S1 to S9
Table S1

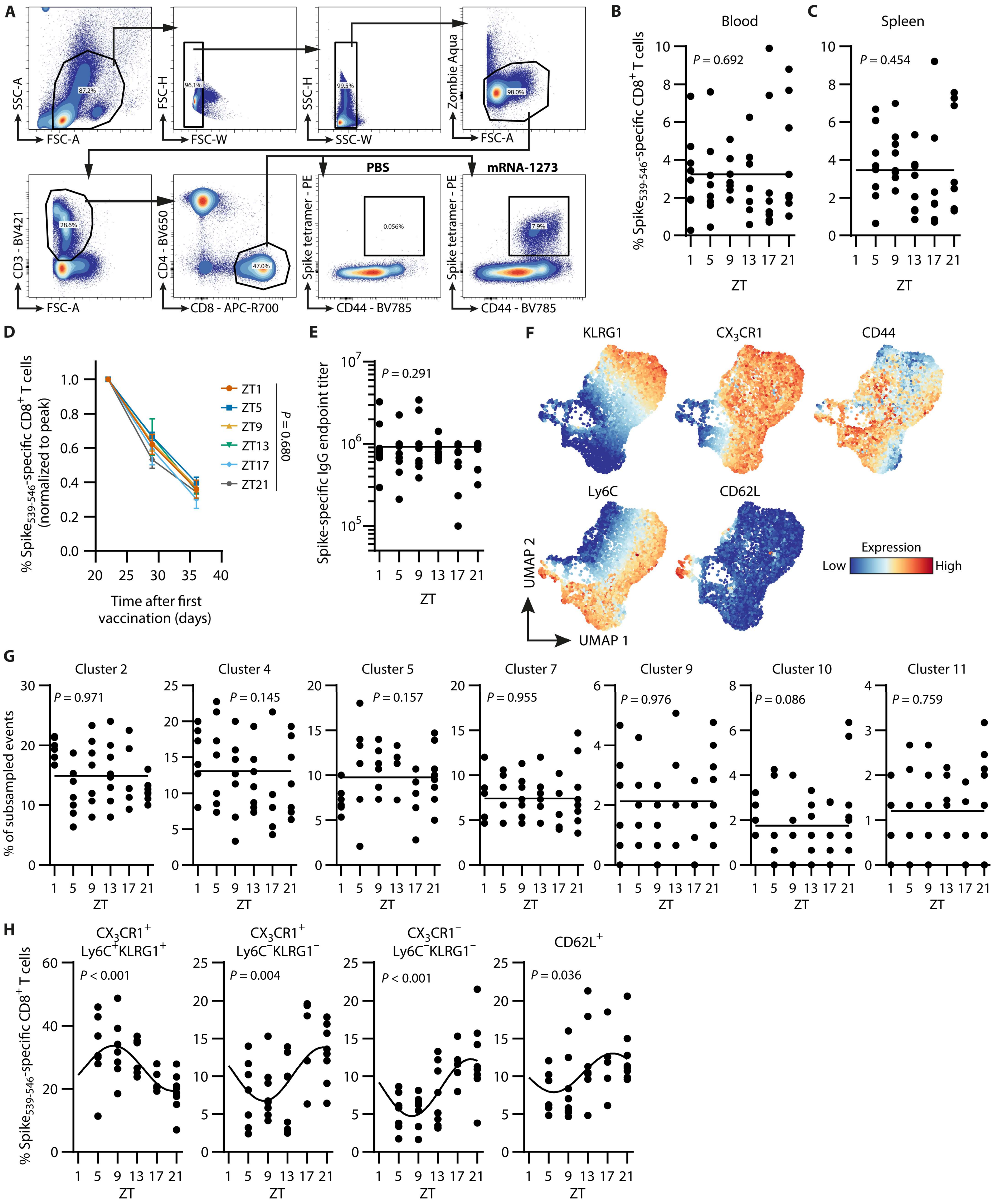
A
SSC-A
FSC-A
87.2%
FSC-H
FSC-W
96.1%
SSC-H
SSC-W
99.5%
Zombie Aqua
FSC-A
98.0%
CD3 - BV421
FSC-A
28.6%
CD4 - BV650
CD8 - APC-R700
47.0%
PBS
Spike tetramer - PE
CD44 - BV785
0.056%
mRNA-1273
Spike tetramer - PE
CD44 - BV785
7.9%
B
Blood
% Spike539-546-specific CD8+ T cells
P = 0.692
ZT
C
Spleen
P = 0.454
ZT
D
% Spike539-546-specific CD8+ T cells (normalized to peak)
Time after first vaccination (days)
ZT1
ZT5
ZT9
ZT13
ZT17
ZT21
P = 0.680
E
Spike-specific IgG endpoint titer
P = 0.291
ZT
F
KLRG1
CX3CR1
CD44
Ly6C
CD62L
Expression
Low
High
UMAP 2
UMAP 1
G
Cluster 2
Cluster 4
Cluster 5
Cluster 7
Cluster 9
Cluster 10
Cluster 11
% of subsampled events
P = 0.971
P = 0.145
P = 0.157
P = 0.955
P = 0.976
P = 0.086
P = 0.759
ZT
H
CX3CR1+ Ly6C+KLRG1+
CX3CR1+ Ly6C−KLRG1−
CX3CR1− Ly6C−KLRG1−
CD62L+
% Spike539-546-specific CD8+ T cells
P < 0.001
P = 0.004
P < 0.001
P = 0.036
ZT

**Fig. S1. Impact of immunization time on expansion, contraction, and differentiation.** (**A**) Representative gating strategy for identification of single live $CD3^+CD4^-CD8^+$ $Spike_{539-546}$-specific cells from blood by p–MHC class I tetramer staining. (**B, C**) Frequency of $Spike_{539-546}$-specific $CD8^+$ T cells in blood (B) or spleen (C) on day 36. (**D**) Frequency of $Spike_{539-546}$-specific $CD8^+$ T cells in blood normalized to the peak of the response (mean ± s.e.m.). (**E**) Spike-specific IgG endpoint titer in serum on day 29. (**F**) Expression intensity of cell surface markers used for UMAP-based dimension reduction (blue, low; red, high). (**G**) Relative abundance of remaining PhenoGraph clusters. (**H**) Frequency of $Ly6C^+CX_3CR1^+KLRG1^+$, $Ly6C^+CX_3CR1^-KLRG1^-$, $Ly6C^-CX_3CR1^-KLRG1^-$, and $CD62L^+$ $Spike_{539-546}$-specific $CD8^+$ T cells in spleen on day 36. For all panels, $n$=8. In (B), (C), (E), (G), and (H), rhythmicity was assessed by cosine fits, with sine waves indicating a significant fit ($P < 0.05$). Statistical analysis was performed using repeated-measures ANOVA in (D). Exact $P$ values are shown on the graph.

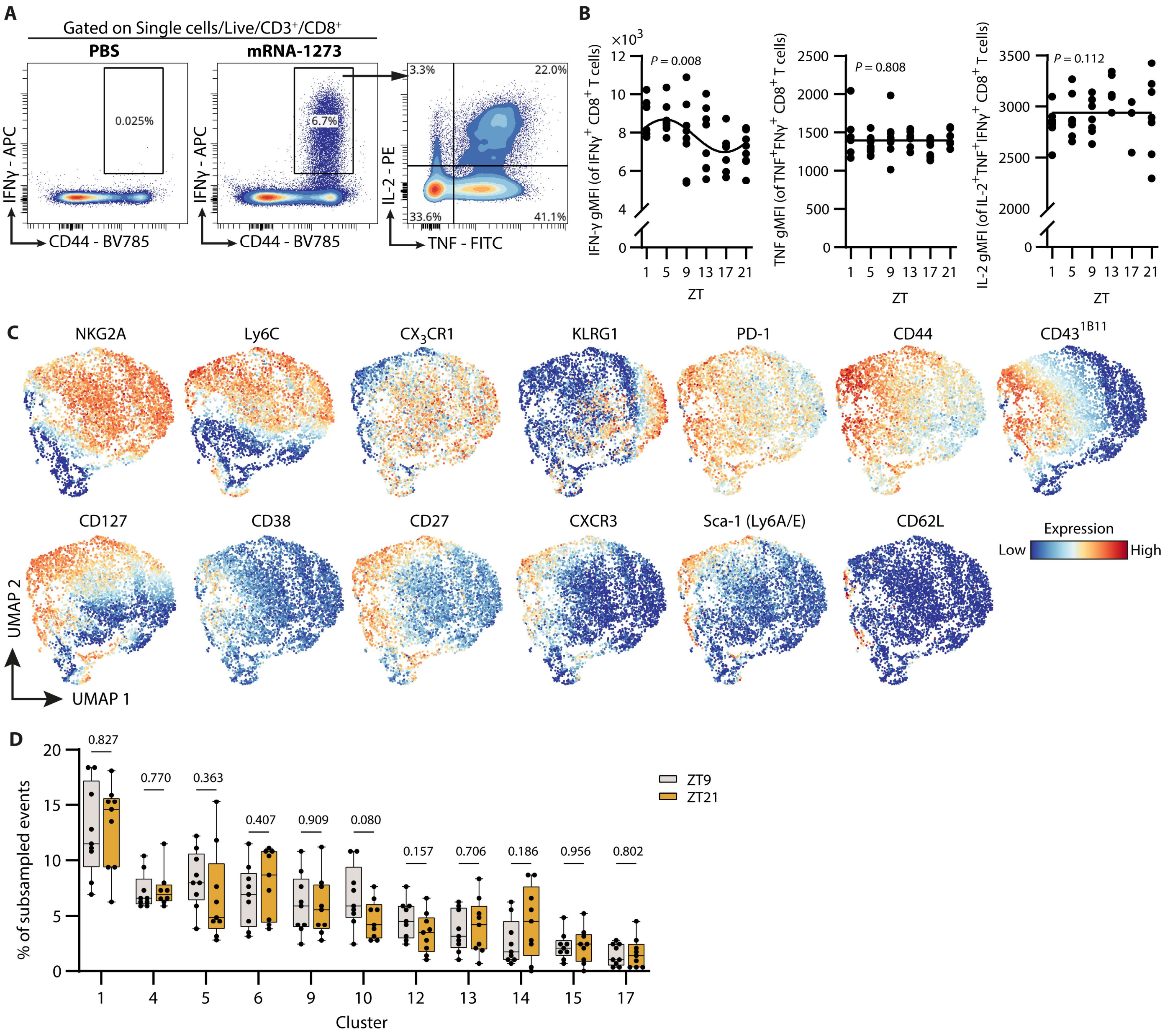


**Fig. S2. In-depth differentiation analysis of Spike539-546-specific CD8⁺ T cells.** (**A**) Representative gating strategy for cytokine-producing cells from the spleen. (**B**) Geometric mean fluorescent intensity (gMFI) of IFN-γ (left), TNF (middle), and IL-2 (right) by cytokine-positive CD8⁺ T cells (*n*=8 per group). (**C**) Expression intensity of cell surface markers used for UMAP-based dimension reduction (blue, low; red, high). (**D**) Relative abundance of remaining PhenoGraph clusters (*n*=8 (ZT9), *n*=9 (ZT21)). In (B), rhythmicity was assessed by cosine fits, with sine waves indicating a significant fit ($P < 0.05$). Statistical analysis was performed using two-sided t-tests in (D). Exact *P* values are shown on the graph.

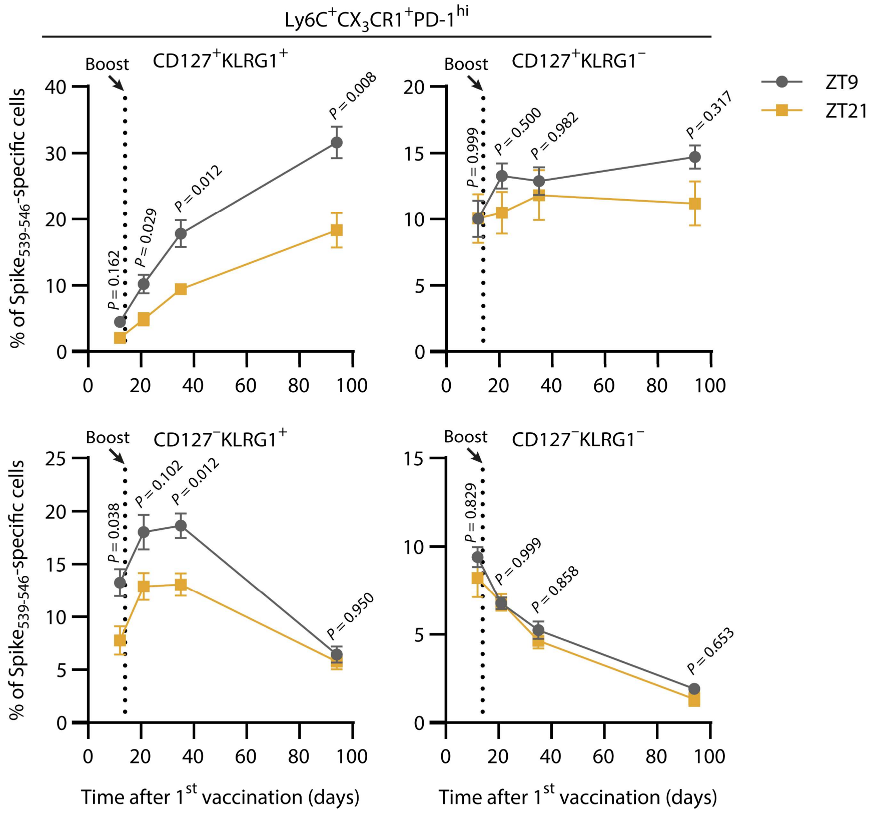


**Fig. S3. CD127 and KLRG1 expression is dynamic during contraction.** Frequency of $CD127^{+}KLRG1^{+}Ly6C^{+}CX_3CR1^{+}PD\text{-}1^{int}$ (top left), $CD127^{+}KLRG1^{-}Ly6C^{+}CX_3CR1^{+}PD\text{-}1^{int}$ (top right), $CD127^{-}KLRG1^{+}Ly6C^{+}CX_3CR1^{+}PD\text{-}1^{int}$ (bottom left), and $CD127^{-}KLRG1^{-}Ly6C^{+}CX_3CR1^{+}PD\text{-}1^{int}$ (bottom right) $Spike_{539\text{-}546}$-specific $CD8^{+}$ T cells in blood (mean ± s.e.m.; *n*=8 (ZT9), *n*=9 (ZT21)). Statistical analysis was performed using two-way repeated-measures ANOVA with Sidak's post hoc test. Exact *P* values are shown on the graph.

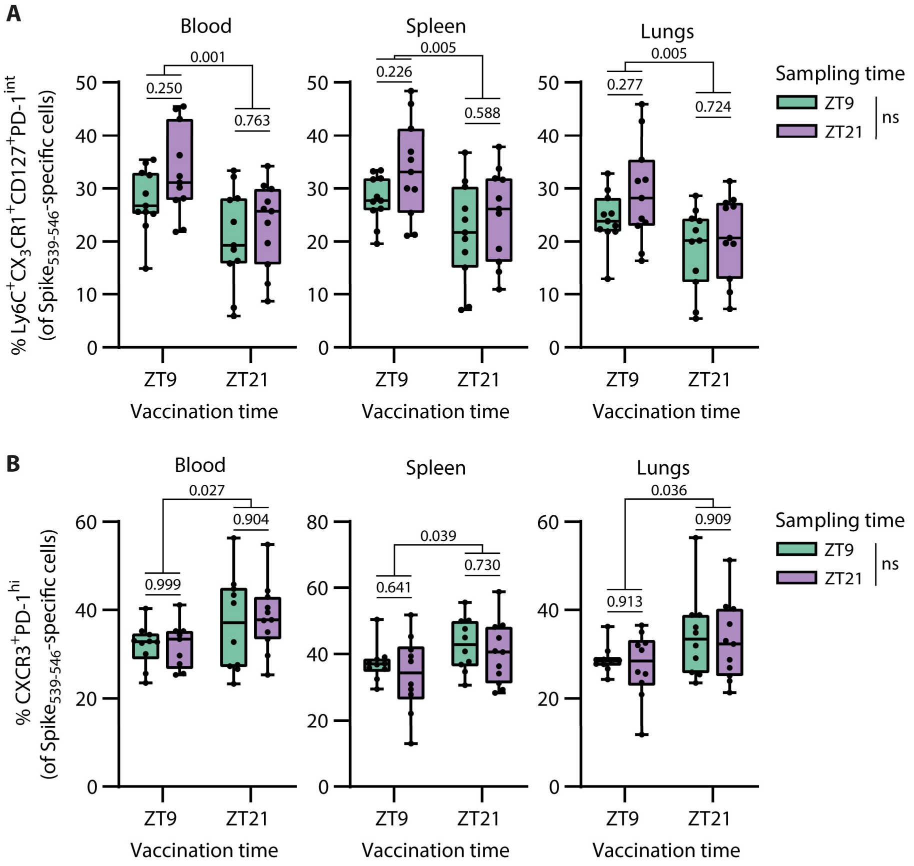


**Fig. S4. Sampling time does not confound CD8$^+$ T cell differentiation phenotypes.** Mice were vaccinated with mRNA-1273 at ZT9 or ZT21 on day 0 and day 14. Sampling was performed 36 days after vaccination for all groups, either at the same ZT as vaccination or with a 12 h offset (36 days + 12 h). (**A, B**) Frequency of Ly6C$^+$CX$_3$CR1$^+$CD127$^+$PD-1$^{int}$ (A) and CXCR3$^+$PD-1$^{hi}$ (B) Spike$_{539-546}$-specific CD8$^+$ T cells ($n$=10 per group). Statistical analysis was performed using two-way ANOVA with Sidak's post hoc test. Exact $P$ values are shown on the graph.

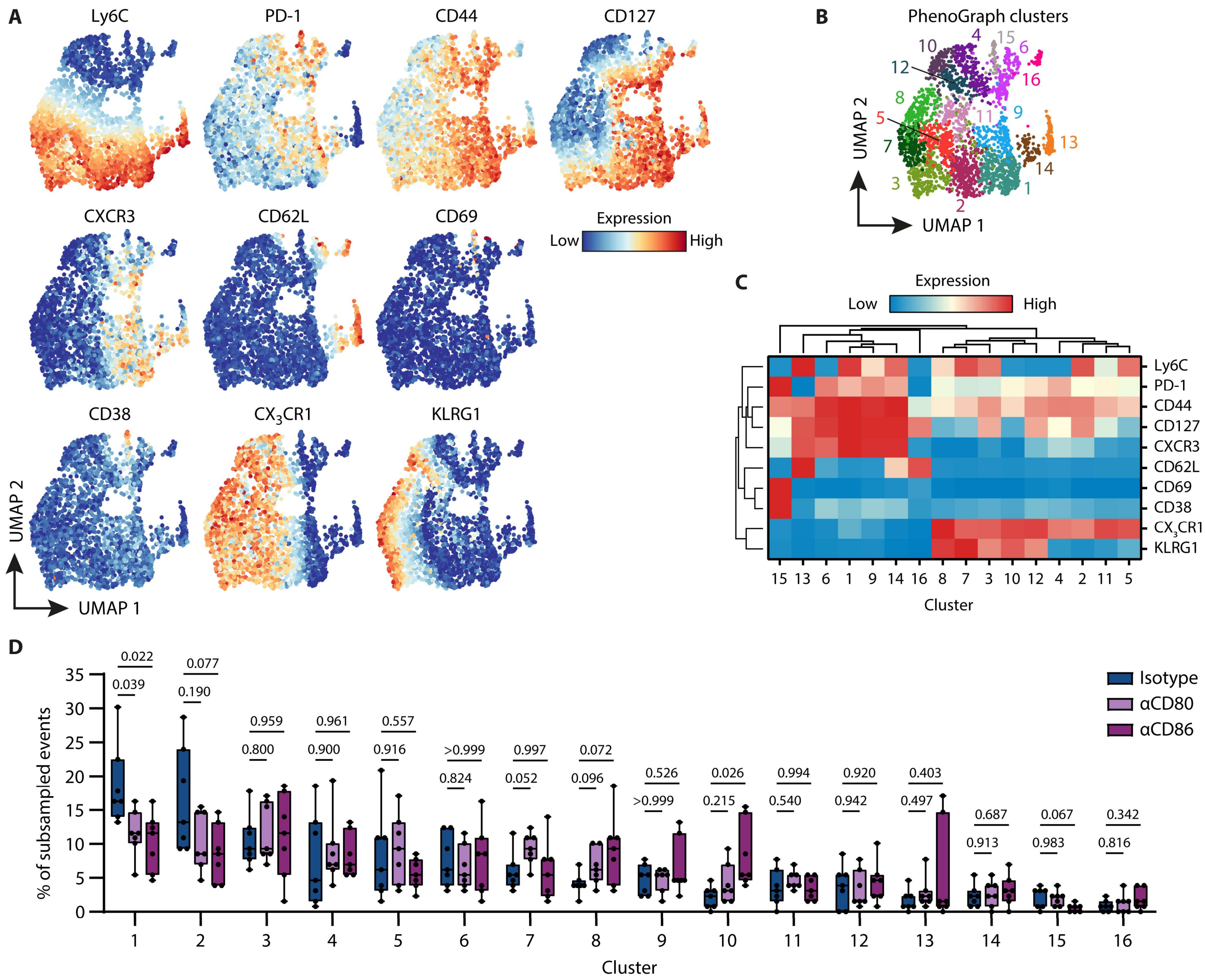


**Fig. S5. Lack of CD80- or CD86-mediated signaling promotes differentiation into CD127⁻ KLRG1⁺ cells.** Dimensionality reduction by UMAP was performed on Spike$_{539\text{-}546}$-specific cells from spleens on day 36 from mice treated with αCD80, αCD86, or isotype control. (**A**) Expression intensity of surface markers used for UMAP-based dimension reduction (blue, low; red, high). (**B, C**) UMAP embedding (B) and corresponding hierarchically clustered heatmap (C) showing normalized marker expression (blue, low; red, high). (**D**) Relative abundance of PhenoGraph clusters (*n*=7 per group). Statistical analysis was performed using two-way repeated-measures ANOVA with Dunnett's post hoc test in (D). Exact *P* values are shown on the graph.

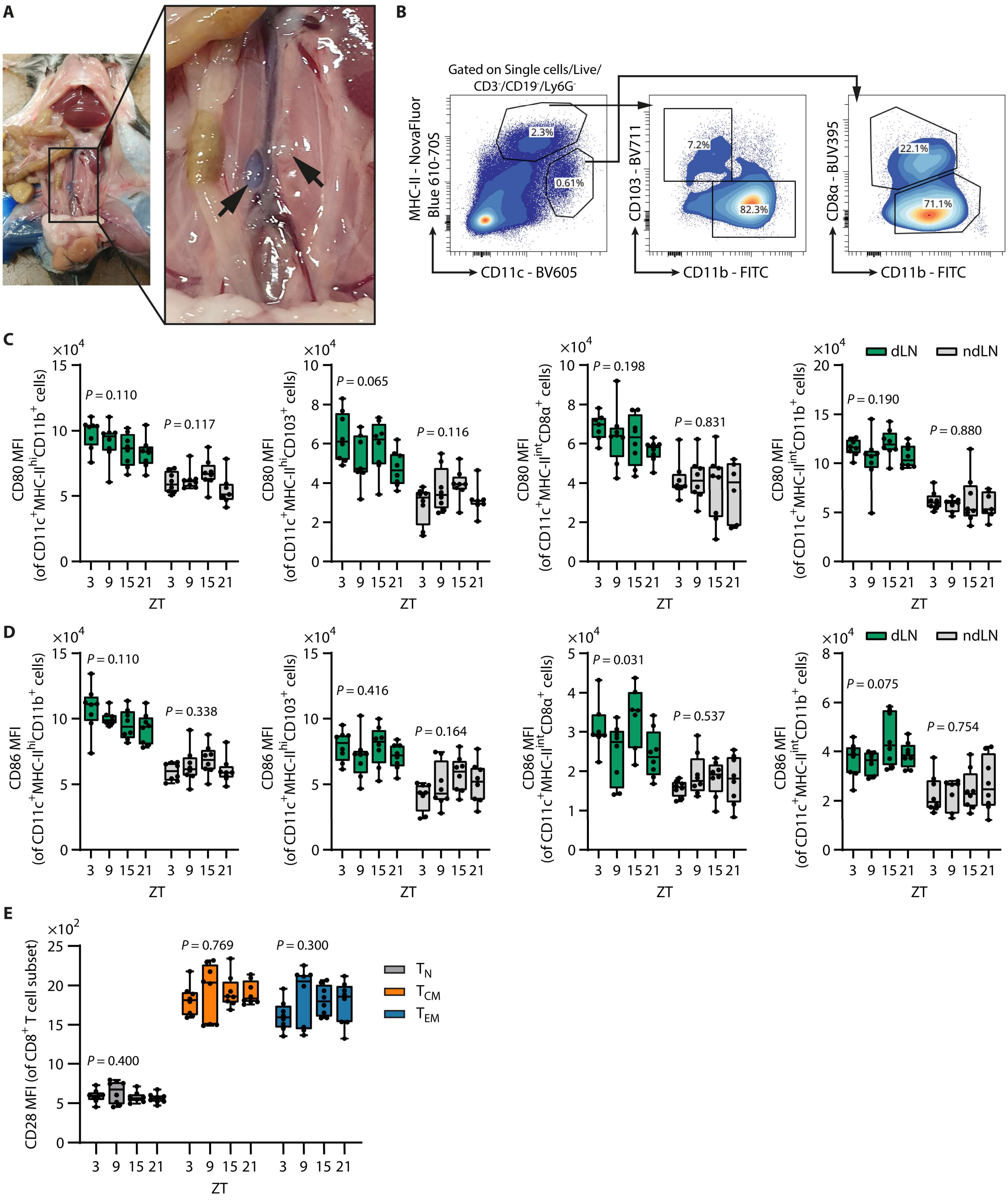


**Fig. S6. Expression of CD80, CD86, and CD28 is arrhythmic.** (**A**) Representative image showing the dLN (left arrow) and ndLN (right arrow) 45 minutes after i.m. injection of 1% methyl

blue in the right hindlimb. (**B**) Representative gating strategy for identification of CD11c$^{+}$MHC-II$^{hi}$ and CD11c$^{+}$MHC-II$^{int}$ DCs in the iliac LN. (**C, D**) Mean CD80 (C) and CD86 (D) expression by CD11c$^{+}$MHC-II$^{hi}$CD11b$^{+}$, CD11c$^{+}$MHC-II$^{hi}$CD103$^{+}$, CD11c$^{+}$MHC-II$^{int}$CD8α$^{+}$, and CD11c$^{+}$MHC-II$^{int}$CD11b$^{+}$ DCs in the dLN and ndLN 48 h after vaccination (*n*=8 per group). (**E**) Mean CD28 expression on $T_N$, $T_{CM}$, and effector memory ($T_{EM}$) CD8$^{+}$ T cells in the dLN 48 h after vaccination (*n*=8 per group). Statistical analysis was performed using one-way ANOVA. Exact *P* values are shown on the graph.

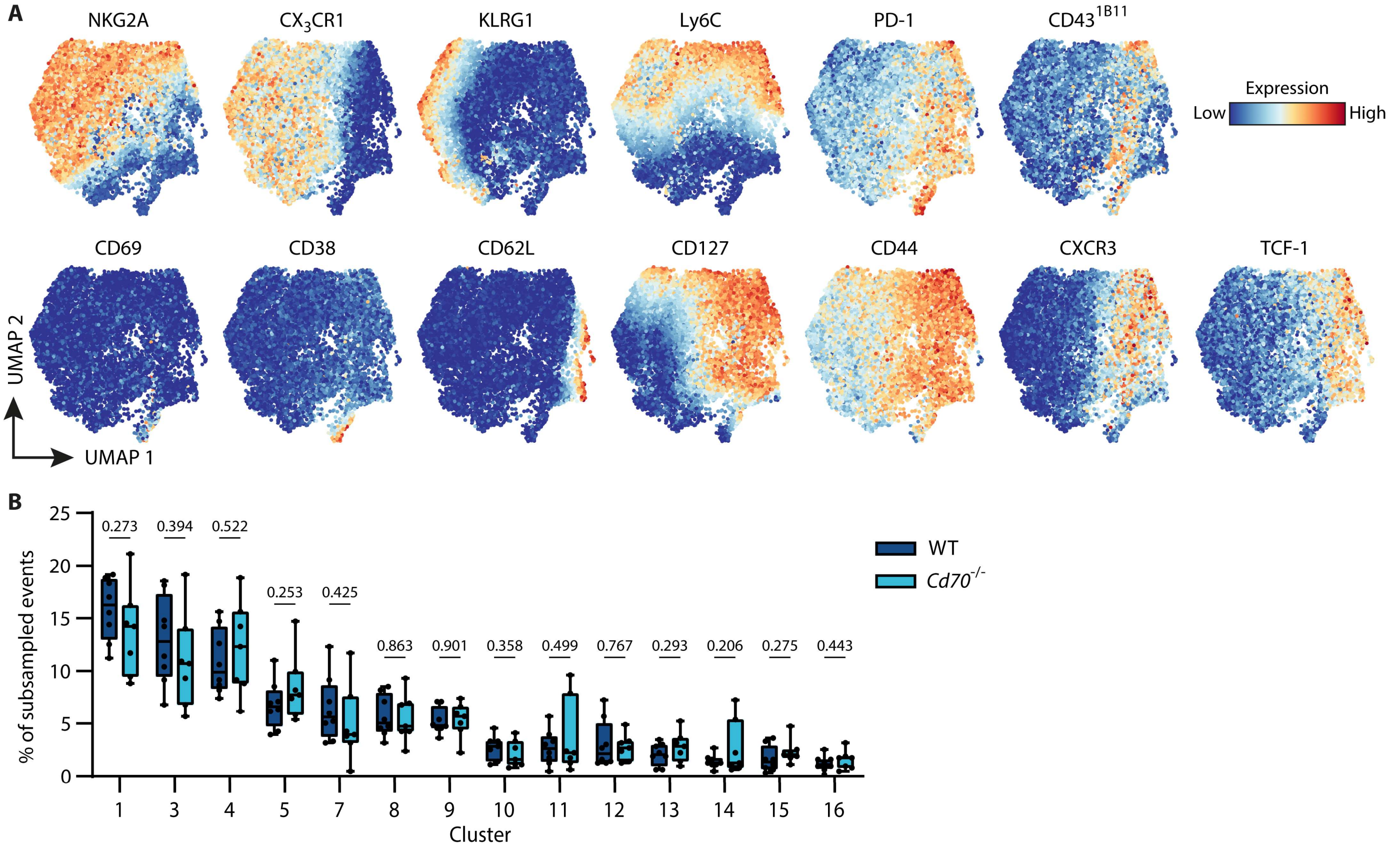


**Fig. S7. In-depth differentiation analysis of *Cd70*$^{-/-}$ Spike$_{539\text{-}546}$-specific CD8$^{+}$ T cells.** (**A**) Expression intensity of markers used for UMAP-based dimension reduction (blue, low; red, high). (**B**) Relative abundance of remaining PhenoGraph clusters (*n*=8 (WT), *n*=7 (*Cd70*$^{-/-}$)). Statistical analysis was performed using two-sided t-tests. Exact *P* values are shown on the graph.

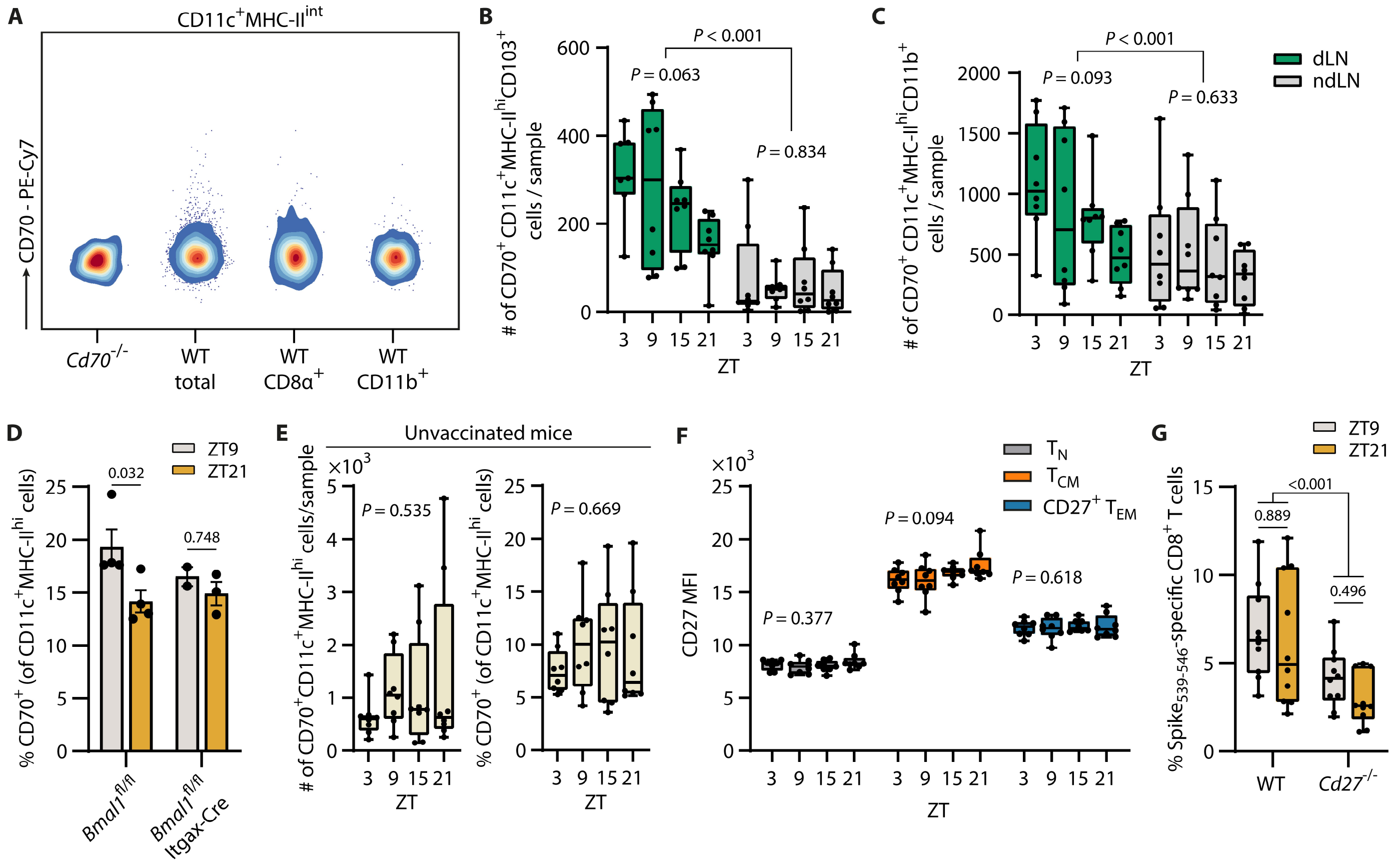


**Fig. S8. Baseline CD70 and CD27 expression is arrhythmic.** (**A**) Representative contour plots showing CD70 expression on CD11c$^+$MHC-II$^{int}$ DCs. (**B, C**) Number of CD70$^+$CD11c$^+$MHC-II$^{hi}$CD103$^+$ (B) and CD70$^+$CD11c$^+$MHC-II$^{hi}$CD11b$^+$ (C) cells 48 h after vaccination (*n*=8 per group). (**D**) CD70 expression on DCs of *Bmal1*$^{fl/fl}$ Itgax-Cre mice and littermate controls (*n*=4 (*Bmal1*$^{fl/fl}$), *n*=2 (*Bmal1*$^{fl/fl}$ Itgax-Cre, ZT9), *n*=3 (*Bmal1*$^{fl/fl}$ Itgax-Cre, ZT21)). (**E**) Number (left) and frequency (right) of CD70$^+$CD11c$^+$MHC-II$^{hi}$ cells in iliac LNs of naïve mice (*n*=8 per group). (**F**) Mean CD27 expression by CD8$^+$ T cells in the dLN 48 h after vaccination (*n*=8 per group). (**G**) Frequency of Spike$_{539\text{-}546}$-specific CD8$^+$ T cells in the spleen on day 36 (*n*=9 (WT, ZT21), *n*=10 (other groups)). Statistical analysis was performed using one-way ANOVA for time effects in (B), (C), (E), and (F), repeated-measures ANOVA for organ effects in (B) and (C), and two-way ANOVA with Sidak's post hoc test in (D) and (G). Exact *P* values are shown on the graph.

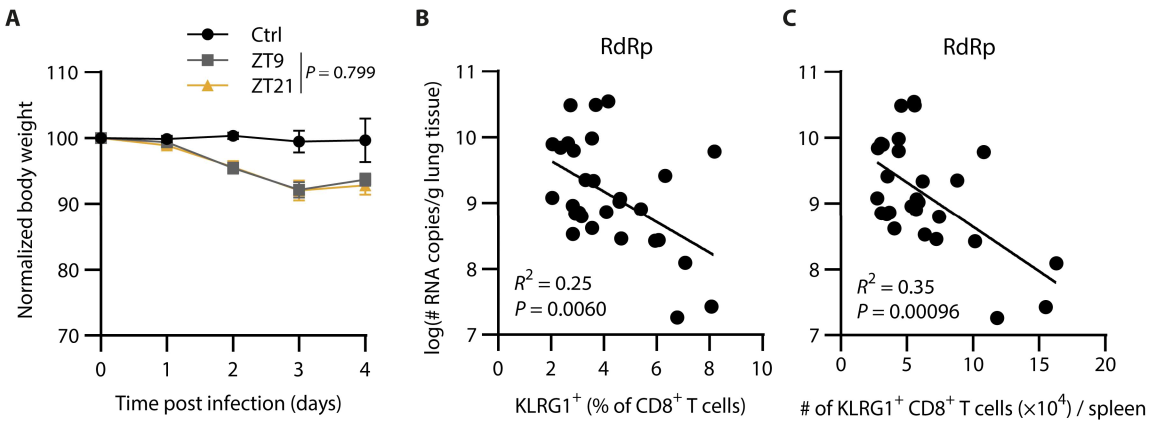


**Fig. S9. Systemic KLRG1$^+$ numbers correlate with improved viral clearance.** (**A**) Normalized bodyweight (day 0 = 100; *n*=4 (control), *n*=14 (other groups)). (**B, C**) Correlation between frequency (B) or number (C) of KLRG1$^+$ CD8$^+$ T cells in the spleen and viral RdRp RNA in the lungs (*n*=28). Statistical analysis was performed using repeated-measures ANOVA in (A) and linear regression with an F test in (B) and (C). Exact *P* values are shown on the graph.

## Table S1. Flow cytometry antibodies

| Target | Fluorophore | Clone | Supplier | Catalog no. | Dilution | RRID |
|---|---|---|---|---|---|---|
| Bcl-2 | PE | 3F11 | BD Biosciences | 556537 | 1:20 | AB_396457 |
| CD3 | BV421 | 17A2 | Biolegend | 100228 | 1:400 | AB_2562553 |
| CD3 | BV711 | 17A2 | Biolegend | 100241 | 1:400 | AB_2563945 |
| CD3 | PE-Fire 700 | 17A2 | Biolegend | 100272 | 1:800 | AB_2876394 |
| CD4 | BV650 | RM4-5 | Biolegend | 100555 | 1:400 | AB_2562529 |
| CD4 | BV711 | RM4-5 | Biolegend | 100550 | 1:200 | AB_2562099 |
| CD4 | PE-Cy7 | RM4-5 | eBioscience | 25-0042-82 | 1:1600 | AB_469578 |
| CD8 | APC | 53-6.7 | Biolegend | 100712 | 1:800 | AB_312751 |
| CD8 | APC-R700 | 53-6.7 | BD Biosciences | 564983 | 1:400 | AB_2739032 |
| CD8 | BUV395 | 53-6.7 | BD Biosciences | 563786 | 1:600 | AB_2732919 |
| CD8 | BV421 | 53-6.7 | Biolegend | 100753 | 1:2000 | AB_2562558 |
| CD8 | FITC | 53-6.7 | Biolegend | 100706 | 1:150 | AB_312754 |
| CD11b | FITC | M1/70 | Biolegend | 101206 | 1:400 | AB_312789 |
| CD11b | Pacific Blue | M1/70 | Biolegend | 101224 | 1:800 | AB_755986 |
| CD11c | BV605 | HL3 | BD Biosciences | 563057 | 1:100 | AB_2737978 |
| CD27 | Alexa Fluor 700 | LG.3A10 | Biolegend | 124240 | 1:200 | AB_2810383 |
| CD27 | PE | LG.3A10 | BD Biosciences | 558754 | 1:200 | AB_397106 |
| CD28 | APC | 37.51 | Biolegend | 102110 | 1:100 | AB_312875 |
| CD38 | BUV737 | 90 | BD Biosciences | 741748 | 1:1600 | AB_2871114 |
| CD43 | PE-Cy7 | 1B11 | Biolegend | 121218 | 1:400 | AB_528813 |
| CD43 | PerCP-Cy5.5 | 1B11 | Biolegend | 121223 | 1:200 | AB_893336 |
| CD44 | BUV805 | IM7 | BD Biosciences | 741921 | 1:800 | AB_2871234 |
| CD44 | BV785 | IM7 | Biolegend | 103059 | 1:400 | AB_2571953 |
| CD62L | BUV395 | MEL-14 | BD Biosciences | 740218 | 1:1600 | AB_3685037 |
| CD62L | BV421 | MEL-14 | Biolegend | 104435 | 1:300 | AB_10900082 |
| CD62L | BV711 | MEL-14 | Biolegend | 104445 | 1:2000 | AB_2564215 |
| CD69 | BUV563 | H1.2F3 | BD Biosciences | 741234 | 1:100 | AB_2870786 |
| CD70 | PE-Cy7 | FR70 | Biolegend | 104611 | 1:200 | AB_2750466 |
| CD80 | FITC | 16-10A1 | eBioscience | 11-0801-85 | 1:400 | AB_465134 |
| CD80 | PE | 16-10A1 | eBioscience | 12-0801-83 | 1:2000 | AB_465753 |
| CD86 | APC | GL1 | eBioscience | 17-0862-82 | 1:2000 | AB_469419 |
| CD86 | BV421 | GL1 | Biolegend | 105032 | 1:400 | AB_2650895 |
| CD103 | BV421 | 2E7 | Biolegend | 121421 | 1:100 | AB_10900074 |
| CD103 | BV711 | 2E7 | Biolegend | 121435 | 1:200 | AB_2686970 |
| CD127 (IL-7Rα) | PE-Cy5 | A7R34 | Biolegend | 135015 | 1:100 | AB_1937262 |
| CD127 (IL-7Rα) | RB780 | A7R34 | BD Biosciences | 569066 | 1:100 | AB_3684748 |
| CD137L (4-1BBL) | Biotin | TKS-1 | eBioscience | 13-5901-85 | 1:200 | AB_466789 |
| CD159 (NKG2A/C/E) | FITC | 20d5 | BD Biosciences | 550520 | 1:200 | AB_393723 |
| CD183 (CXCR3) | APC-Fire 750 | CXCR3-173 | Biolegend | 126540 | 1:100 | AB_2650829 |
| CD186 (CXCR6) | BV711 | SA051D1 | Biolegend | 151111 | 1:50 | AB_2721558 |
| CD252 (OX40L) | Biotin | RM134L | Biolegend | 108804 | 1:200 | AB_313403 |
| CD279 (PD-1) | PE-Dazzle594 | 29F.1A12 | Biolegend | 135228 | 1:1000 | AB_2566005 |

| | | | | | | |
|---|---|---|---|---|---|---|
| $CX_3CR1$ | APC-Fire 810 | SA011F11 | Biolegend | 149053 | 1:1600 | AB_2910300 |
| $CX_3CR1$ | BV785 | SA011F11 | Biolegend | 149029 | 1:1600 | AB_2565938 |
| $CX_3CR1$ | PE | SA011F11 | Biolegend | 149006 | 1:400 | AB_2564315 |
| I-A/I-E (MHC-II) | NovaFluor Blue 610-70S | M5/114.15.2 | eBioscience | M024T02B06 | 1:400 | AB_2910831 |
| IFN-γ | APC | XMG1.2 | eBioscience | 17-7311-82 | 1:200 | AB_469504 |
| IL-2 | PE | JES6-5H4 | eBioscience | 12-7021-82 | 1:200 | AB_466150 |
| Ki-67 | BV605 | 16A8 | Biolegend | 652413 | 1:800 | AB_2562664 |
| KLRG1 | BV605 | 2F1 | Biolegend | 138419 | 1:300 | AB_2563357 |
| KLRG1 | BV785 | 2F1 | Biolegend | 138429 | 1:100 | AB_2629749 |
| KLRG1 | PE-Cy7 | 2F1 | Biolegend | 138416 | 1:100 | AB_2561736 |
| Ly6A/E (Sca-1) | BUV496 | D7 | BD Biosciences | 750169 | 1:1000 | AB_2874374 |
| Ly6C | APC-Cy7 | AL-21 | BD Biosciences | 560596 | 1:400 | AB_1727555 |
| Ly6C | BV605 | HK1.4 | Biolegend | 128035 | 1:400 | AB_2562352 |
| Ly6C | BV785 | HK1.4 | Biolegend | 128041 | 1:1600 | AB_2565852 |
| Ly6C | Pacific Blue | HK1.4 | Biolegend | 128013 | 1:600 | AB_1732079 |
| TNF | FITC | MP6-XT22 | Biolegend | 506304 | 1:1000 | AB_315425 |
| TNF | PE-Cy7 | MP6-XT22 | Biolegend | 506323 | 1:400 | AB_2204356 |
